# Discovering Age- and Sex-Specific Genetic Risk Factors in Sensorineural Hearing Loss: Genome-Wide Evidence from Large-Scale Biobanks

**DOI:** 10.1007/s10162-026-01054-y

**Published:** 2026-05-26

**Authors:** Argyro Bizaki-Vallaskangas, Eeva Sliz, Tuuli Lankinen, Elmo Saarentaus, Ville Salo, Kristi Krebs, Tytti Willberg, Ilkka Kivekäs, Joel Rämö, Sanna Toppila-Salmi, Aarno Dietz, Vesa Hytönen, Aarno Palotie, Lili Milani, Antti Mäkitie, Johannes Kettunen

**Affiliations:** 1 Faculty of Medicine and Health Technology, Department of Otolaryngology, University of Tampere, Tampere, Finland; 2 Research Unit of Population Health, Biocenter Oulu, and Medical Research Center Oulu, University of Oulu and Oulu University Hospital, Oulu, Finland; 3 Faculty of Medicine, University of Helsinki, Helsinki, Finland; 4 Institute for Molecular Medicine Finland (FIMM), HiLIFE, University of Helsinki, Helsinki, Finland; 5 Estonian Genome Centre, Institute of Genomics, University of Tartu, Tartu, Estonia; 6 Department of Otolaryngology, Turku University Hospital, Turku, Finland; 7 Department of Otorhinolaryngology, University of Eastern Finland, Joensuu and Kuopio, Finland, Wellbeing services county of Pohjois-Savo, Kuopio, Finland; 8 Department of Allergology, Inflammation Center, Helsinki University Hospital and University of Helsinki, Helsinki, Finland; 9 Department of Medicine and Health Technology, University of Tampere, Finland and Fimlab Laboratories, Tampere, Finland; 10 The Stanley Center for Psychiatric Research and Program in Medical and Population Genetics, The Broad Institute of MIT and Harvard, Cambridge, MA USA; 11 Department of Medicine, Department of Neurology and Department of Psychiatry Massachusetts General Hospital, Analytic and Translational Genetics Unit, Boston, MA USA; 12 Department of Otorhinolaryngology – Head and Neck Surgery, Helsinki University Hospital and University of Helsinki, Helsinki, Finland; 13 Research Program in Systems Oncology, Faculty of Medicine, University of Helsinki, Helsinki, Finland; 14 Department of Otorhinolaryngology, Tampere University Hospital, Tampere, Finland

**Keywords:** FinnGen, Sensorineural hearing loss, SNHL, GWAS, Inner ear, Genetics

## Abstract

**Purpose:**

Investigate the genetic components of sensorineural hearing loss (SNHL) by performing genome-wide meta-analyses using the data from FinnGen and Estonian Biobank.

**Methods:**

We studied genome-wide associations of SNHL in FinnGen and the Estonian Biobank in the general population and in sex- and age-of-onset stratified subgroups. The study-specific GWASs were combined through inverse variance-weighted genome-wide meta-analyses, encompassing a total of 531,059 individuals (Ncases = 35,960). Age-stratified meta-analyses included 28,198 individuals diagnosed at the age of 55 years or after and 7762 individuals diagnosed before the age of 55 years, with 495,099 controls. Sex-stratified meta-analyses included 313,501 females (Ncases = 17,761) and 217,558 males (Ncases = 18,199).

**Results:**

In the meta-analysis focusing on the general population, 22 SNHL-associated loci (±1 Mb window) were observed, three of which were previously unreported. In the sex-stratified analysis, two previously unreported SNHL loci were observed in the female subgroup and one locus in the male subgroup. Additionally, in the age-stratified analysis, two previously unreported SNHL loci were observed in the subgroup of those that were diagnosed at the age of 55 years or after. In those diagnosed before the age of 55 years, one previously unreported locus was observed. Overall, 32 loci were associated with SNHL at *p* < 5 × 10^–8^ in at least one of the study groups. Of these, nine have not been previously reported in association with SNHL. We also estimated if there were significant differences in the effect sizes of the lead variants at each locus between the analytical subgroups and observed differences for 14 variants.

**Conclusions:**

Previously unreported SNHL risk loci and differences in effect sizes found in this study provide additional insight into the genetic underpinnings of SNHL. Our results validate the role of mechano-transduction and genetic components affecting the structure of the inner ear in the background of SNHL. Our study contributes to our understanding of the genetic causes of SNHL and may open the door for further translational research.

**Supplementary Information:**

The online version contains supplementary material available at 10.1007/s10162-026-01054-y.

## Introduction

Sensorineural hearing loss (SNHL) is the most common sensory deficit affecting individuals across all ages. It may have genetic or environmental origins and can present as either syndromic, occurring alongside other clinical features, or non-syndromic, where hearing loss is the sole manifestation. SNHL results from damage to the hair cells of the inner ear or the vestibulocochlear nerve. While hair cell damage is a major contributor to SNHL, there are also other mechanisms, such as synaptopathy or stria vascularis dysfunctions, that may lead to SNHL. Presbyacusis, or age-related hearing loss (ARHL), is the most prevalent form of hearing loss, which affects approximately 35% of those aged 65–74 years and 50% of those aged 75 and older [1, 2]. It is usually characterized by progressive bilateral high-frequency hearing loss, which impairs speech recognition, especially in noisy environments [1–3]. The quality of life can be severely impacted by hearing loss, necessitating significant changes to daily routines. SHNL is also a major disability that causes significant economic costs and is linked to severe comorbidities, such as dementia, depression, and social isolation, all of which can further worsen quality of life [4–7].

Hearing loss has a significant genetic component, with heritability estimates ranging typically 35–65%, being up to 70% for age-related hearing impairment (ARHI) in twin studies [8–13]. Previous genome-wide association studies (GWASs) have identified numerous loci associated with SNHL and defined the complex nature of SNHL [14–28]. Due to varying definitions of the phenotypes, studies in isolated populations [17, 18], and overall modest sample sizes, some of these earlier hearing loss-related GWAS have lacked consistency. Because of these factors and the high degree of genetic heterogeneity in hearing loss, the findings have not been consistently replicated in following studies [22]. Clinical studies have shown that a patient’s sex might affect the presentation time and severity of SNHL, but the underlying genetic basis is still poorly understood. Despite the high prevalence of SNHL in the aging population, only a few age-stratified GWASs have been conducted.

Owing to these shortcomings, the present study was designed to identify age of onset or sex-specific loci with stratified GWAS analyses. Here, we present the results of genome-wide meta-analyses of SNHL using the data from FinnGen and the Estonian Biobank. The meta-analysis identified novel SNHL loci, and sex- and age-stratified analyses revealed additional loci, with some variants showing differential effect estimates across strata.

## Materials and Methods

### Study Populations

FinnGen is a collaborative project launched in 2017 [29], aiming at advancing human health through genetic research. This initiative harnesses genome data collected from a nationwide network of Finnish biobanks, which are linked with digital health records from various national registries, including hospital discharge, death, cancer, and medication reimbursement databases. The project covers approximately 10% of the Finnish population. FinnGen Data Freeze 9 data utilized in the study included 27,232 SHNL cases and 326,172 controls (Table S3).

The Estonian Biobank (EstBB) is a longitudinal biobank in Northern Europe that holds genotype and phenotype data for 20% of the Estonian adult population [30]. At recruitment, participants signed a consent allowing follow-up linkage of their electronic health records (EHR). Health records at EstBB are extracted from the data provided by regional hospitals, national registries, and the Estonian Health Insurance Fund, a national administrative database that pools detailed and individual-level billing data for all health care services as well as digital prescription information. The activities of the EstBB are regulated by the Human Genes Research Act, which was adopted in 2000 specifically for the operations of the EstBB. Individual-level data analysis in the EstBB was carried out under ethical approval number 1.1–12.1/624 from the Estonian Committee on Bioethics and Human Research (Estonian Ministry of Social Affairs). In this study, 177 655 participants from EstBB were included, 8728 cases and 168 927 controls (Table S3).

### Phenotype Descriptions

We identified SNHL cases that were required to have an entry of *The International Classification of Diseases, Tenth Revision* (ICD-10): H90.3, H90.4, H90.5, H91.1. Participants without a record of these ICD codes were classified as controls. In addition, the individuals with the records of the following codes were excluded from the analyses: H65.4, H66.1, H66.2, H66.3, H67.0*A18.6, H67.0*A38, H67.1*, H67.1*B05.3, H68.0, H68.1, H70.0, H70.1, H70.2, H70.8, H70.9, H71, H73.1, H73.8, H73.9, H74.0, H74.1, H74.2, H74.3, H74.4, H74.8, H74.9, H75.0*, H75.0*A18.0, H75.8*, H80.0, H80.1, H80.20, H80.28, H80.8, H80.9, H81.0, H83.0, H83.1, H83.2, H83.3, H83.8, H83.9, H90.0, H90.1, H90.2, H90.6, H90.7, H90.8, H91.0#, H91.2, H91.3, H91.8, H91.9, H93.3, H93.8, H93.9, H94.0*, H94.0*A52.1, H94.8*, H95.0, H95.1, H95.8, H95.9, Q16.0, Q16.1, Q16.2, Q16.3, Q16.4, Q16.5, Q16.9, Q17.2, Q17.3, Q17.4, Q17.8, Q17.9, D33.30. Overall, the meta-analysis focusing on the general population consisted of 35,960 SNHL cases and 495,099 controls (Table S3).

**Fig. 1:**
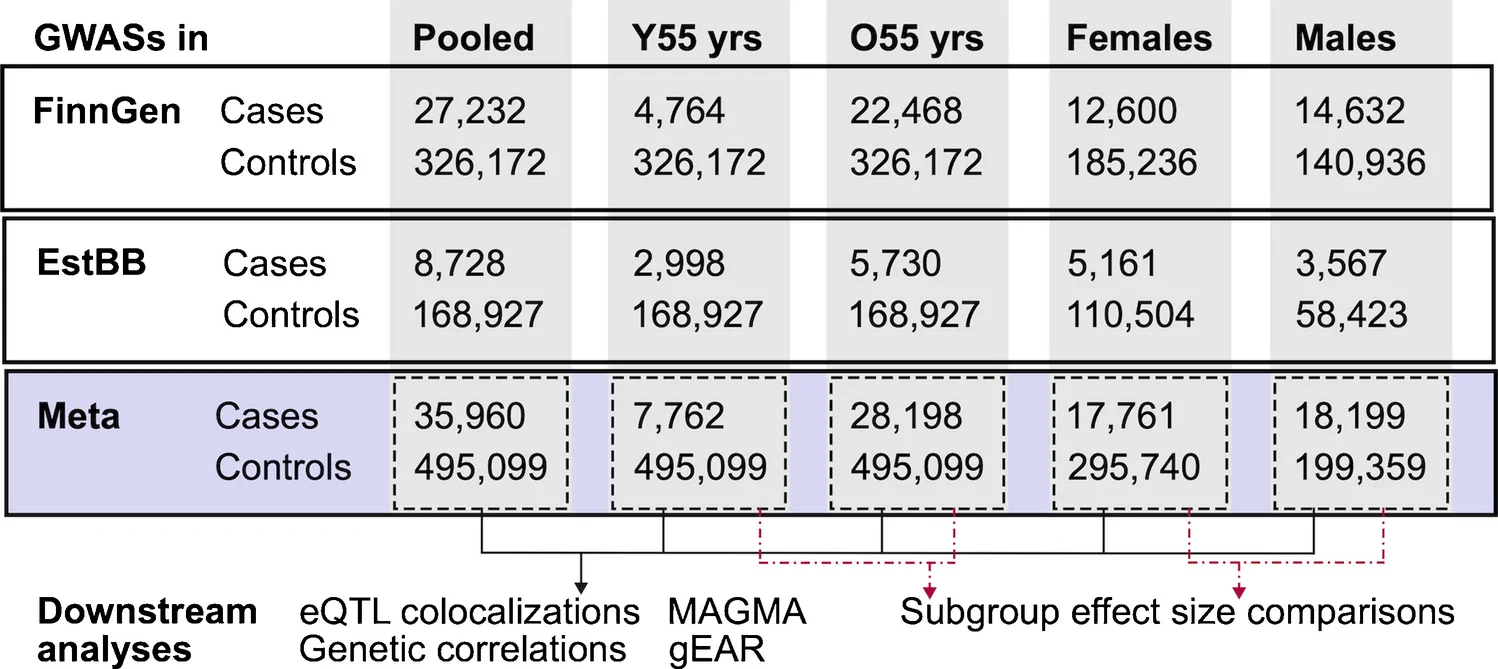
Overview of the study design for the SNHL GWAS. The schematic illustrates the datasets used and the subsets analyzed in the study

In the sex-stratified analyses, participants were stratified to males (18,199 cases in the meta-analysis) and females (17,761 cases in the meta-analysis) and were compared to same-sex controls (the respective numbers of controls were 199,359 and 295,740). In the age-stratified analyses, the cases were classified into two groups: those who were diagnosed before the age of 55 (7762 cases) and those who were diagnosed at the age of 55 or after (28,198 cases). Earlier criteria for age-related hearing loss often used 60 years as the cut-off age, whereas more recent recommendations suggest considering screening around age 50 [31]; therefore, we selected 55 years as a threshold that better reflects the recent recommendations while remaining within the commonly reported onset range of approximately 55–65 years [32]. The control group was not stratified by age in the age-stratified analyses (N_controls_ = 495,099 in the age-of-onset-stratified analysis). The number of participants in each studied cohort is available in Table S3 and in the study overview given in Fig. 1.

### Genotyping, Imputation, and Quality Control

In FinnGen, genotyping of the samples was performed using Illumina and Affymetrix arrays (Illumina Inc., San Diego, and Thermo Fisher Scientific, Santa Clara, CA, USA). Sample quality control (QC) was performed to exclude individuals with high genotype missingness (>5%), ambiguous gender, excess heterozygosity (±4SD) and non-Finnish ancestry. Regarding variant QC, all variants with low Hardy–Weinberg equilibrium (HWE) *p*-value (< 1e-6), high missingness (>2%) and minor allele count (MAC) < 3 were excluded. Chip genotyped samples were pre-phased with Eagle 2.3.5, with the number of conditioning haplotypes set to 20,000. Genotype imputation was conducted by using the Finnish population-specific SISu v3 reference panel with Beagle 4.1 (version 08Jun17.d8b) as described in the following protocol: dx.doi.org/10.17504/protocols.io.nmndc5e. In post-imputation QC, variants with imputation INFO<0.6 were excluded.

Genotyping of the samples from the Estonian Biobank was conducted in the Core Genotyping Lab of the Institute of Genomics, University of Tartu, using the following Illumina arrays: GSAMD-24v1, GSAMD-24v2, ESTchip-1_GSAv2-MD, and ESTchip-2_GSAv3-MD. Quality control was conducted according to best practices (exclusion of individuals with call rate < 95%, with a mismatch between genotype and phenotype sex and who deviated ±3SD from the samples heterozygosity rate mean; exclusion of SNVs with call rate < 95%, HWE p < 1e-4, MAF < 1%). Samples were pre-phased with Eagle v2.3 with the number of conditioning haplotypes set to 20,000, and genotype imputation was carried out with Beagle v.28Sep18.793 using an Estonian-specific reference panel [33]. Prior to imputation, variants with minor allele frequency (MAF) <1% and indels were removed. Further, EstBB samples were combined with the 1000 genomes phase 3 dataset for ancestry analysis. Genetic principal components were calculated using a subset of quality-controlled and pruned genotyped SNPs. This was further used to identify and remove samples that deviated from the main cluster.

### GWASs and Meta-analyses

GWASs were conducted with REGENIE (v2.2.4) using an additive model [34]. The analyses were adjusted for age, sex, and the first 10 genetic principal components, and in FinnGen, also for the genotyping batch. In the sex-stratified analyses, sex was not included as a covariate.

The METAL software (v.2011-03-25) [35] was used to do inverse-variance weighted fixed-effect meta-analyses of the GWAS results from FinnGen and the Estonian Biobank. Variant data from the Estonian Biobank were converted from hg19 to hg38 prior to the meta-analysis. A total of 14,848,072 genetic variants available through both FinnGen and EstBB were included in the meta-analysis.

### Characterization of the Association Signals

We defined a locus as a window of 1 MB (±500,000 bases from the association lead variant) containing at least one variant associated with SNHL at *P* < 5 × 10^–8^. By manually annotating every gene within the loci and prioritizing those according to a relevant biological function using literature and database resources such as Genbank [36] and Uniprot [37], we were able to identify potential candidate genes for the loci that had not been linked to SNHL in previous studies.

### Functional Annotations

We employed MAGMA v1.10 (Linux) [38] for conducting gene-based and gene-set analysis on SNHL data. The input file utilized in the analysis comprised meta-analyzed results from the SNHL GWAS conducted in the general population. The 1000 Genomes project European population was used as a reference sample for LD corrections. The variants were mapped to gene locations according to human genome build 38 (NCBI38.gene.loc). To better capture variants in the regulatory regions, we added a 35 kb window upstream and a 10 kb window downstream of the genes. Subsequently, the association signals of the variants were aggregated to gene-level statistics. The gene-set level analysis used the 2023.2 version of the MSigDB gene set file, comprising 34,494 curated gene sets and gene ontology (GO) terms.

### eQTL Colocalizations

Gene expression and observed SNHL associations were colocalized using the “coloc” R-library function ‘coloc.abf’ [39]. For every SNHL-associated locus, colocalization was evaluated for every gene within a 1 MB window from the association lead variant. From the GTExportal (https://www.gtexportal.org/home/datasets), we downloaded variant-gene expression associations (GTEx v8, accessed on 19/05/2022) for the analysis. We selected the following tissues for the analysis: whole blood as a widely used reference tissue; brain (cerebellum, cortex, and hippocampus) and tibial nerve to assess alignment with neuronal expression relevant to auditory signaling; and adipose tissue (subcutaneous and visceral) to capture regulatory variation related to metabolic and inflammatory processes that may contribute to ARHL. We considered posterior probabilities for a shared causal variant ≥ 0.8 as a limit for significant results [40].

To gain a better understanding of the gene expression of potential candidate genes in tissues relevant to SNHL, we reviewed gene expression profiles for 24 identified potential candidate genes from the gEAR [41], using the Human inner ear atlas datasets [42]. We did not identify potential candidate genes for loci located in the HLA region, so no genes from the region were included in the gene expression analysis.

### Genetic Correlations

Genetic correlations were computed using the LDSC software [43]. Genetic correlations between SNHL and 767 other traits were computed using data taken from the MRC Integrative Epidemiology Unit (IEU)’s GWAS database (https://gwas.mrcieu.ac.uk/). The significance limit for correlations was set at a P_FDR_ < 0.05.

### Subgroup Effect Differences

We estimated if there were significant differences in the effect sizes of the lead variants at each locus between the analytical subgroups (i.e., those diagnosed before the age of 55 years vs. those diagnosed at or after 55 years of age, and females vs. males) using the following formula, with the corresponding *P*-values calculated from the normal distribution:

$${Z}_{diff}=|{beta}_{group1}-{beta}_{group2}|/\sqrt{({se}_{group1}^{2}+{se}_{group2}^{2})}$$

Effect size differences with P_FDR_ < 0.05 were deemed statistically significant.

We used five prediction tools for assessing the consequences of the missense mutations (Table 1). PolyPhen-2 [44, 45], Sorting Tolerant From Intolerant (SIFT) [46, 47], and Protein Variation Effect Analyzer (PROVEAN) [48] assess the consequences of the mutation using conservation-based prediction, evaluating the variants by comparing similar proteins and considering local sequence conservation. To utilize meta-prediction and machine learning, we used the Rare Exome Variant Ensemble Learner (REVEL) [49], which has been shown to improve the prediction accuracy compared to methods based on sequence conservation alone. OpenCRAVAT interface was utilized in this approach (https://www.opencravat.org/), and we also report the consensus among all the missense prediction tools included in that platform. We exploited AlphaMissense, which combines structural context and evolutionary conservation of the mutated position to predict the consequences of missense variants [50, 51].

**Table 1:** Prediction of the consequences of the missense variants with selected algorithms. The prediction and scoring are provided for all the predictors. Unfortunately, AlphaMissense prediction was not available for rs2242416. Consensus score obtained from OpenCRAVAT is reported, where the number refers to predictions reporting the mutation being benign or tolerated; the total number of predictors applied is shown as well

rsid/Gene	Mutation	SIFT		PolyPhen-2 HDIV		PROVEAN		REVEL		CRAVAT	AlphaMissense	
rs146694394 *SYNJ2*	T656M	Damaging	0.001	Probably damaging	1.000	Deleterious	−5.504	Uncertain	0.655	2/13	Likely pathogenic	0.730
rs2242416 *CRIP3*	I188S	Tolerated	0.397	Possibly damaging	0.793	Neutral	−0.060	Uncertain	0.473	4/11	N/A	N/A
	I188T	Tolerated	0.397	Benign	0.093	Neutral	1.39	Uncertain	0.314	8/11	N/A	N/A
	I188N	Tolerated	0.304	Probably damaging	0.982	Neutral	−0.870	Uncertain	0.598	3/11	N/A	N/A
rs5756795 *TRIOBP*	F1187I	Damaging	0.023	Possibly damaging	0.678	Neutral	−2.15	Benign	0.102	8/11	Likely benign	0.258
	F1187L	Tolerated	0.089	Benign	0.006	Neutral	−2.02	Benign	0.042	11/11	Likely pathogenic	0.745

## Results

### Loci Associated with SNHL in General Population

**Fig. 2:**
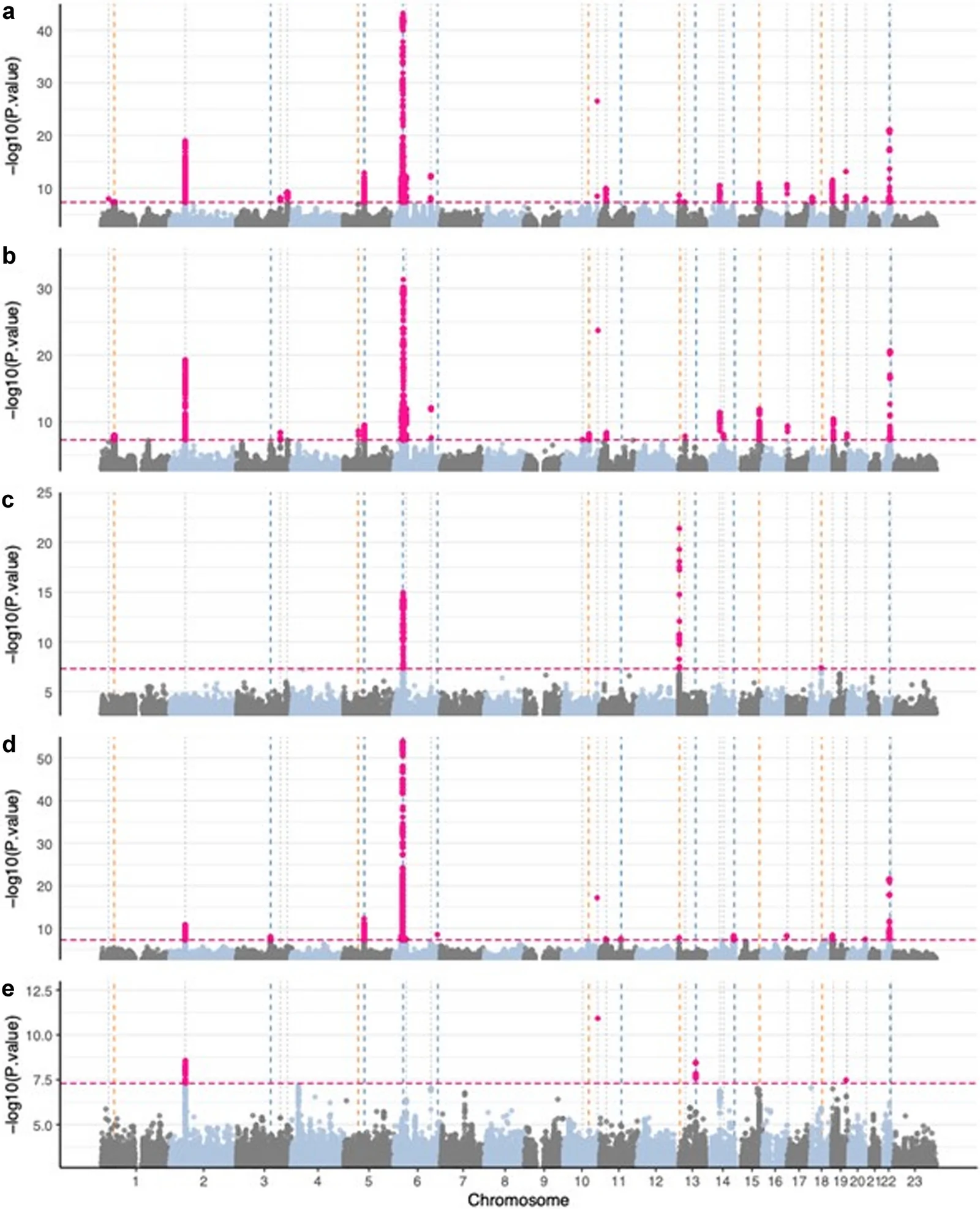
Manhattan plots of the SNHL associations in **a** the general population, **b** 55 years or older, **c** younger than 55, **d** females, and **e** males. Vertical dashed lines indicate loci that were associated with SNHL with genome-wide significance (*P* < 5 × 10^–8^) in at least one of the analytical subgroups; color coding is as follows: orange = FDR-significant effect size difference in o55 vs. y55; blue = pFDR-significant effect size difference in females vs. males; gray = no FDR-significant effect size differences

An overview of the study design is shown in Fig. 1. In our genome-wide meta-analyses of SNHL, we identified 22 genome-wide significant (*P* < 5 × 10^–8^) loci associated with SNHL (Fig. 2A, Figure S1, Table S3). Of these, three loci near genes *COG6* (component of oligomeric golgi complex 6), *PIEZO1* (piezo type mechanosensitive ion channel component 1) and *CDH4* (cadherin 4) had not been reported in association with SNHL in prior studies (Table 2, regional plots Figure S2-S4), whereas the remaining 19 were located proximally to known SNHL risk loci (Table S3) [15, 20, 21, 23, 25, 27, 28, 52]. The association signals at 6p21.31-6p22.2 located in the *HLA* region extend beyond the 1 Mb locus definition and were counted as one locus. Although the FinnGen and the Estonian Biobank results were generally consistent, we observed some heterogeneity in the complex associations at the HLA and 19q13.33 loci (Table S3-S7). Only a few of the identified variants were more common in Finnish and Estonian population compared to European allele frequencies, for example rs1297538889 variant near *PIEZO1* (EAF_Meta_ = 0.002, EAF_FinnGen_ = 0.003, EAF_EstBB_ = 0.001 EAF_European_ = 7.4 × 10^–5^, Table S3-S7).

**Table 2:** Novel SNHL-associated genome-wide significant (*P* < 5 × 10^–8^) loci identified in the genome-wide meta-analyses based on FinnGen and the Estonian Biobank. A full list of SNHL-associated genome-wide significant loci identified in the genome-wide meta-analyses can be seen in Table S4–Table S8

General population									
Locus	Gene	CHR:POS	rsid	EA	OA	***P***-value	Cohort	OR (95% CI)	EAF
13q14.11	***COG6***	13:39970434	rs9532483	T	G	4.40 × 10^–8^	All	1.05 (1.03–1.07)	0.71
						1.43 × 10^–8^	O55	1.06 (1.04–1.08)	
						0.169	Y55	1.03 (0.99–1.06)	
						5.17 × 10^–4^	Females	1.05 (1.02–1.07)	
						1.86 × 10^–5^	Males	1.06 (1.03–1.08)	
16q24.2	***PIEZO1***	16:88498571	rs1297538889	A	G	2.03 × 10^–11^	All	1.69 (1.54–1.85)	0.002
						4.20 × 10^–10^	O55	1.74 (1.57–1.92)	
						0.018	Y55	1.57 (1.21–1.94)	
						6.04 × 10^–9^	Females	1.89 (1.68–2.10)	
						1.34 × 10^–4^	Males	1.54 (1.32–1.76)	
20q13.33	***CDH4***	20:61270238	rs78892715	T	C	9.10 × 10^–9^	All	1.08 (1.05–1.10)	0.11
						2.33 × 10^–5^	O55	1.07 (1.04–1.09)	
						3.02 × 10^–6^	Y55	1.13 (1.08–1.18)	
						3.25 × 10^–8^	Females	1.11 (1.07–1.14)	
						0.006	Males	1.05 (1.02–1.09)	
Age-stratified analysis: 55 or older									
5q11.1	***ISL1***	5:51156672	rs116773267	A	G	2.27 × 10^–9^	O55	1.12 (1.08–1.16)	0.93
						1.18 × 10^–7^	All	1.09 (1.06–1.12)	
						0.701	Y55	1.01 (0.95–1.08)	
						9.62 × 10^–5^	Females	1.10 (1.05–1.14)	
						2.87 × 10^–4^	Males	1.09 (1.04–1.14)	
10q22.1	***COL13A1***	10:69811134	rs12772368	T	C	3.99 × 10^–8^	O55	1.07 (1.05–1.10)	0.16
						3.71 × 10^–7^	All	1.06 (1.04–1.08)	
						0.360	Y55	1.02 (0.98–1.06)	
						3.18 × 10^–4^	Females	1.06 (1.03–1.09)	
						1.52 × 10^–4^	Males	1.06 (1.03–1.09)	
Age-stratified analysis: Under 55									
18q12.3	***LINC01901***	18:39882914	rs182243786	T	A	3.81 × 10^–8^	Y55	1.62 (1.37–1.93)	0.01
						4.23 × 10^–4^	All	1.19 (1.08–1.31)	
						0.415	O55	1.05 (0.93–1.18)	
						0.069	Females	1.14 (0.99–1.)	
						0.003	Males	1.23 (1.07–1.42)	
Sex-stratified analysis: Females									
11q13.5	***MYO7A***	11:77210313	rs12793619	G	A	2.64 × 10^–8^	Females	1.07 (1.05–1.09)	0.62
						2.04 × 10^–6^	All	1.04 (1.02–1.06)	
						5.80 × 10^–4^	O55	1.03 (1.02–1.05)	
						2.66 × 10^–4^	Y55	1.06 (1.03–1.10)	
						0.313	Males	1.01 (0.99–1.04)	
14q32.23	***KLC1***	14:103725580	rs7143885	C	G	5.02 × 10^–9^	Females	1.07 (1.05–1.10)	0.40
						5.83 × 10^–5^	All	1.03 (1.02–1.05)	
						6.32 × 10^–5^	O55	1.04 (1.02–1.06)	
						0.103	Y55	1.03 (0.99–1.06)	
						0.901	Males	1.00 (0.98–1.02)	
Sex-stratified analysis: Males									
13q31.1	***POU4F1***	13:78781316	rs756330	G	A	3.45 × 10^–9^	Males	1.12 (1.08–1.16)	0.09
						0.001	All	1.05 (1.02–1.07)	
						0.013	O55	1.04 (1.01–1.07)	
						0.070	Y55	1.05 (1.00–1.11)	
						0.173	Females	0.97 (0.94–1.01)	

### Age-Specific Results

We identified 20 genome-wide significant (*P* < 5 × 10^–8^) loci within the subgroup of SNHL cases diagnosed at the age of 55 or older, with two of these loci near *ISL1* (*ISL LIM homeobox 1*), and *COL13A1* (*collagen type XIII alpha 1 chain*) not previously associated to SNHL (Fig. 2B, Table 2, Figures S5-S6, Table S4). In the subgroup of SNHL cases diagnosed before the age of 55, we observed three genome-wide significant loci, of which the one near *LINC01901* (*long intergenic non-protein coding RNA 1901*) was novel (Fig. 2C, Table 2, Figure S7, Table S5). Two of the 15 genome-wide significant loci observed in the female subgroup have not previously been associated with SNHL, these loci located near *MYO7A* (*myosin VIIA*) and *KLC1* (*kinesin light chain 1*) (Fig. 2D, Table 2, Figure S8-S9, Table S6). Four genome-wide significant loci were identified from the male subgroup, and the one near *POU4F1* (*POU class 4 homeobox 1*) was novel (Fig. 2E, Table 2, Figure S10, Table S7).

### Effect Differences

**Fig. 3:**
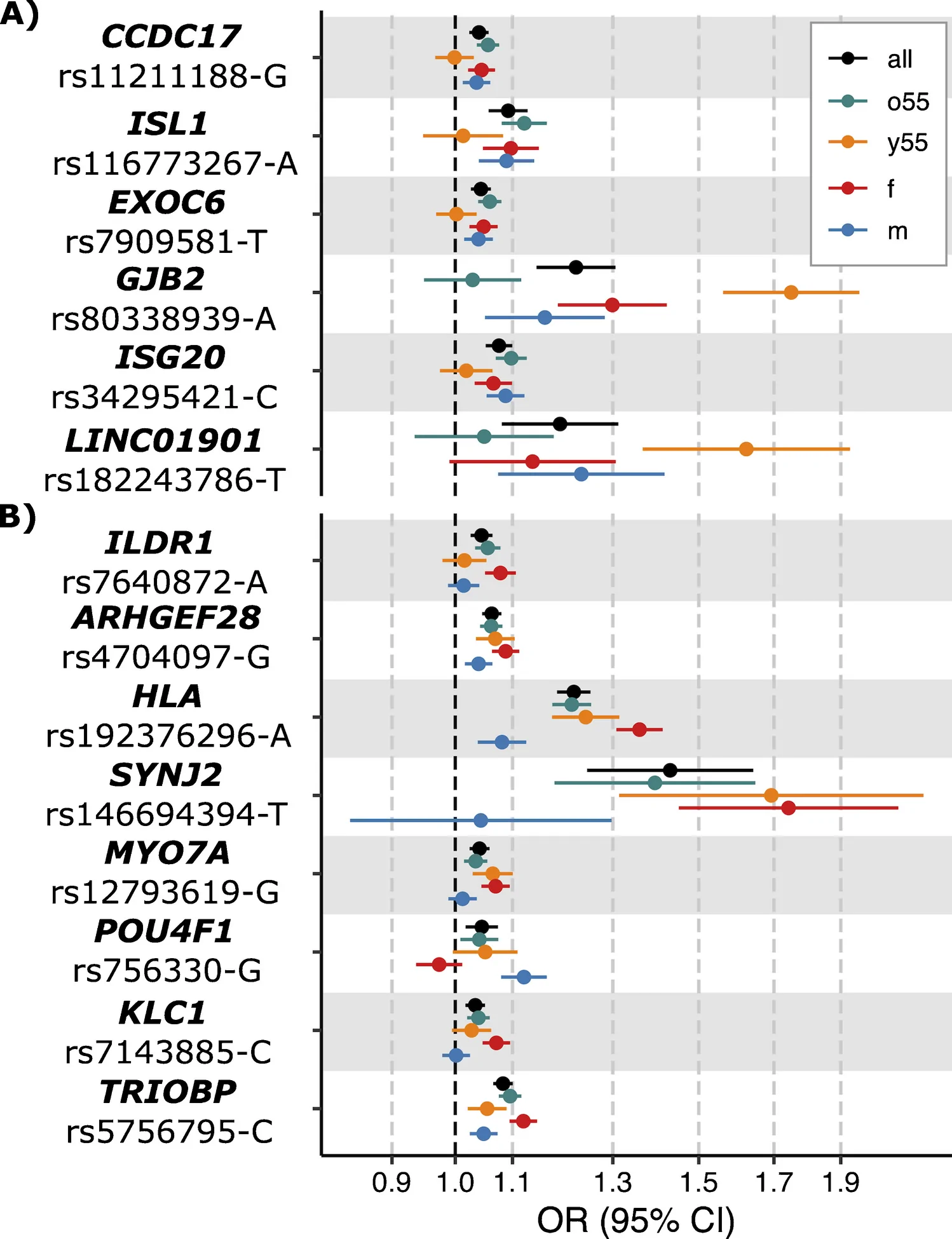
Significant differences in the effect sizes of the lead variants at the SNHL-associated loci between **A** the age-of-onset-stratified subgroups and **B** sex-stratified subgroups. Variants are identified by candidate gene, rsid, and SNHL risk-increasing allele. General population [all, black]; older than 55 years [o55, green]; younger than 55 years [y55, orange]; females [f, red]; males [m, blue]

We observed significant age differences in effect sizes for lead variants near *CCDC17* (*coiled-coil domain containing 17*), *ISL1*, *EXOC6* (*exocyst complex component 6*), *GJB2* (*gap junction protein beta 2*), *ISG20* (*interferon stimulated exonuclease gene 20*), and 18q12.3 locus, rs182243786, which was classified as *LINC01901* (Fig. 3A). Additionally, significant sex differences in effect sizes were observed for lead variants near *ILDR1* (*immunoglobulin like domain containing receptor 1*), *ARHGEF28* (*Rho guanine nucleotide exchange factor 28*), *HLA* rs192376296, *SYNJ2* (*synaptojanin 2*), *MYO7A*, *POU4F1*, *KLC1*, and *TRIOBP* (*TRIO and F-actin binding protein*) (Fig. 3B).

### MAGMA, Inner Ear Gene Expression, and eQTL Colocalizations

**Fig. 4:**
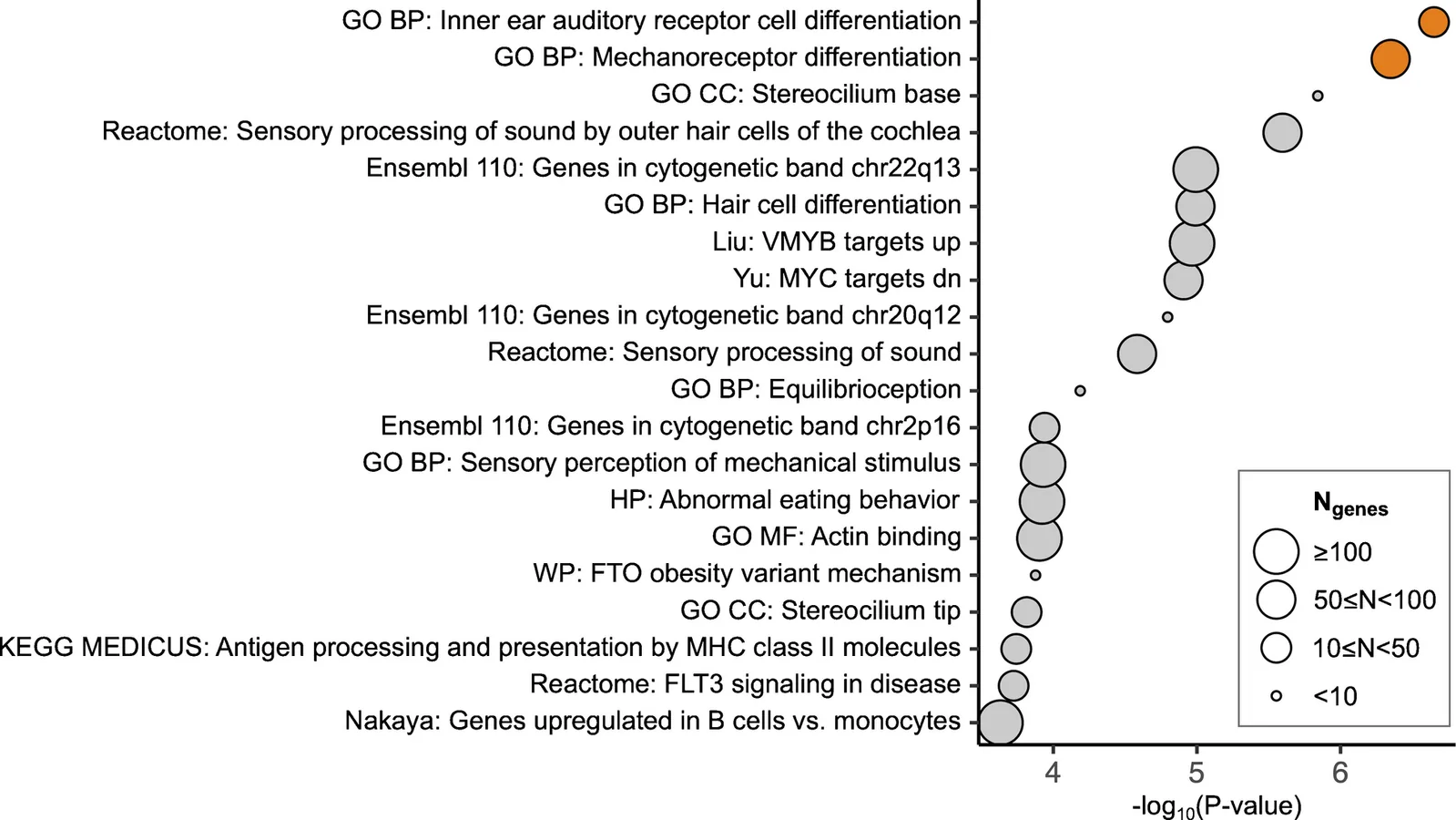
MAGMA gene-set analysis. We conducted MAGMA gene-set analysis using 34,494 curated gene sets and Gene Ontology (GO) terms from the MSigDB [77] (version 2023.3). The plot displays the 20 most significant gene sets, with two highlighted in orange, indicating significance following false discovery rate (FDR) correction

Overall, we identified 78 genes significantly associated with SNHL in the MAGMA gene-based test (*P* < 5.04 × 10^–7^ to correct for 19,823 genes and five subgroups; Table S8). The MAGMA gene-set analysis revealed the most significant enrichments in the pathway related to inner ear auditory receptor cell differentiation (*P* = 2.24 × 10^–7^, P_FDR_ = 0.039) and mechanoreceptor differentiation (*P* = 4.48 × 10^–7^, P_FDR_ = 0.039) in the full sample (Fig. 4). Gene-set analyses conducted in the remaining analytical subgroups did not yield results significant after multiple testing correction (Figure S12).

In a screening of potential candidate gene expression using gEAR, we observed that several genes were expressed in all ear cell types examined (Figure S11). Particularly strong average expression levels were observed for *GAS2* (*growth arrest specific 2*) in several cell types. Hair cells were distinctive among the cell types because the majority of the potential candidate genes were expressed there. For example, *MYO7A*, *TRIOBP* (*TRIO and F-actin* binding protein), and *SYNJ2* (*synaptojanin 2*) had the strongest expressions in hair cells compared to all cell types (Figure S12).

Colocalizations between SNHL GWAS and gene expression signals (eQTL) were observed in every tissue studied (Figure S12). The expression of the 102 genes and SNHL signals was found to colocalize in at least one tissue (posterior probability [PP] for the shared variant ≥ 0.8). For example, SNHL association signals and expression of *CCDC163* (*CCDC163 homolog*) and *SPTBN1* (*spectrin beta, non-erythrocytic 1*) were colocalized in six out of the seven tissues under investigation (Figure S13).

### Genetic Correlation Analysis Reveals Extensive Links Between SNHL and Hearing-Related Traits

**Fig. 5:**
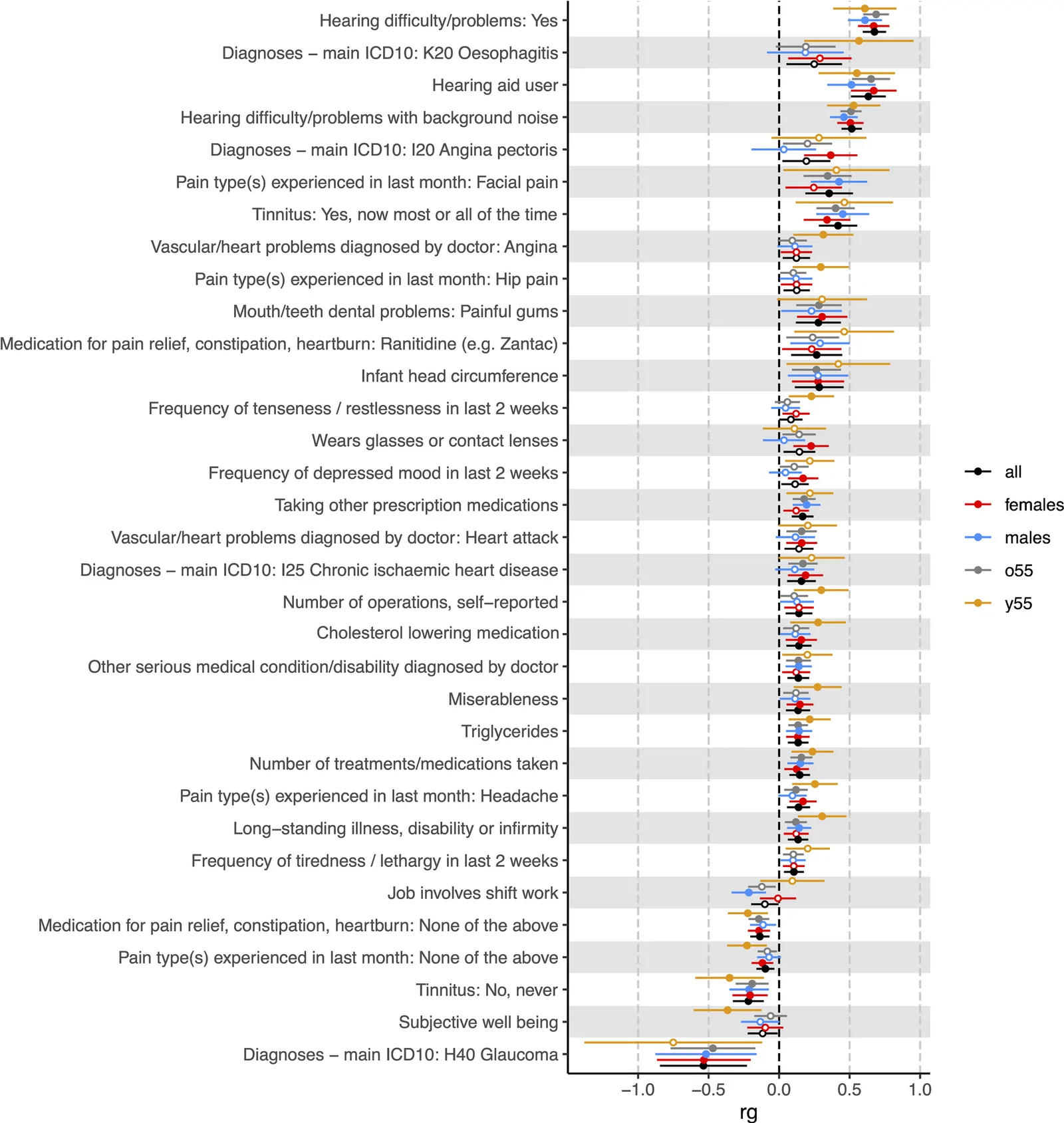
Genetic correlations between SNHL and other traits. Genetic correlations (rg) were calculated using LDSC-software [43], SNHL summary statistics from the general population (all), and from each analyzed subgroup (females, males, older than 55 [o55], younger than 55 [y55]). Summary statistics for the comparison traits were obtained from the GWAS database provided by the MRC Integrative Epidemiology Unit (IEU) (https://gwas.mrcieu.ac.uk/). The plot shows correlations with absolute rg > 0.2 reaching false discovery rate-corrected p-value [pFDR] < 0.05 in at least one of the studied subgroups. Fill/no fill indicates statistical significance (pFDR < 0.05). The full set of results is provided in Table S9

We tested genetic correlations of SNHL with 767 other traits. The strongest positive correlations between SNHL and other traits were observed with “hearing difficulty/problems: Yes” and “hearing aid user” (rg = 0.68 and rg = 0.63, respectively, in the full sample, Fig. 5, Table S10). Also, we observed positive correlations with other hearing-related traits such as “hearing difficulty/problems with background noise” (rg = 0.53 in the full sample, Fig. 5) and “tinnitus: Yes, now most or all of the time” (rg = 0.42 in the full sample, Fig. 5). Additionally, a strong negative correlation that reached the significance threshold was observed with “H40: Glaucoma” (ukb-d-H40, rg = −0.54 in the full sample), but since the result did not replicate with another glaucoma endpoint (bbj-a-121, rg = −0.02 in the full sample), this result requires further investigation before firm conclusions can be drawn. Overall, genetic correlations were largely consistent across the study groups, although coefficients tended to be larger in the subgroup diagnosed before age 55 (Fig. 5). In males, a significant negative genetic correlation with the occupational trait “Job involves shift work” (rg = −0.21) was observed in males only, whereas the correlation did not reach significance in other subgroups (Fig. 5).

In the prediction of the consequences for missense variants, *SYNJ2* variant rs146694394 indicated the most evident influence on protein structure–function (Table 1). For *CRIP3* rs2242416, the predictions were somewhat dependent on the exact deviation in the sequence, all of the variant types showing rather mild effects. Similarly, while modest, the *TRIOBP* variant rs5756795 showed mild effects on protein. AlphaMissense predictions correlated poorly with the other prediction results.

## Discussion

Twenty-two SNHL-associated loci were associated in the genome-wide meta-analysis of the general population (35,960 cases and 531,059 controls), three of which were previously unreported. Furthermore, three previously unreported loci were observed in the sex-stratified analyses and three in the age-stratified analyses, totaling nine previously unreported SNHL risk loci observed in this study. We also replicated multiple loci previously implicated in Mendelian forms of hearing loss and associated with sensorineural hearing loss in prior studies. These include well-known risk loci in or near *SYNJ2*, *TRIOBP*, *GJB2* (gap junction protein beta 2), as well as *MAST2*, *SPTBN1*, *ILDR1*, *ARHGEF28*, and *NID2* [22, 27, 53].

### Previously Unreported SNHL Risk Loci

We detected previously unreported loci near genes that play a role in mechanotransduction, such as *MYO7A* and *PIEZO1*. *MYO7A* is a non-conventional actin-based motor protein required for hair cell development and maturation. In the cochlea, *MYO7A* localizes to sensory hair cell stereocilia, where it participates in tip-link–associated complexes essential for mechanotransduction [54, 55]. Pathogenic variants in *MYO7A* cause both syndromic and non-syndromic forms of sensorineural hearing loss, including Usher syndrome type 1B, DFNA11 and DFNB2 [50, 56]; however, to our knowledge, our genome-wide meta-analysis is the first to report an association between *MYO7A* and sensorineural hearing loss at the population level. Also, *PIEZO1* is proposed to have a role in mechanotransduction [57]. As mechanotransduction converts sound-induced mechanical energy into neuronal signals, it is a fundamental process for hearing [58], and defects in this pathway may contribute to the pathophysiology of sensorineural hearing loss. Histopathological analyses of the aging human cochlea have demonstrated a significant age-related decline in stereocilia regularity and an increase in stereocilia loss, changes that likely contribute to age-related hearing threshold elevations [59]. In addition, we observed previously unreported SNHL associations near *ISL1* and *POU4F1* that potentially regulate differentiation and maintenance of inner ear sensory neurons [60]. No protein-coding genes were located at one of the identified loci; however, the association at this locus may be explained by *LINC01901*, a long intergenic non-coding RNA with currently unknown molecular function and no established link to hearing loss. Its identification in our age of onset-stratified analysis provides evidence that regulatory non-coding elements may contribute to risk of sensorineural hearing loss.

### From Association to Function

Several of the same genes that we had annotated to be potential candidate genes were identified in the MAGMA gene-based analysis, supporting our interpretation. In the MAGMA gene-set analysis, enrichments were observed in the pathways related to the structure of the stereocilia and its differentiation, such as stereocilium base and inner ear auditory receptor cell differentiation. These results were in line with previously reported results, which have reported similar enrichments for different gene sets related to the structures of the inner ear [25, 27]. We observed genetic correlations between a number of different hearing-related traits, which is consistent with shared underlying biology and supports the validity of our analyses. Also, these results were strongly in line with previously reported genetic correlations [20]. Based on consequence prediction performed for missense variants, it is likely that none of the variants cause complete inactivation of the target protein.

### Subgroup Differences

In the age of onset-stratified analysis, effect sizes were significantly larger in SNHL cases diagnosed before the age of 55 for lead variants near *GJB2* and *LINC01901*, while effects of lead variants in or near *CCDC17*, *ISL1*, *ISG20*, and *EXOC6* were larger among patients diagnosed at the age of 55 or older. The stronger effects of the *GJB2* variant in younger individuals are consistent with the well-established role of this gene in hereditary and early onset hearing loss [61, 62]. In contrast, variants showing larger effects in older individuals may reflect genetic influences on age-related degeneration of cochlear hair cells or auditory neurons, which are key mechanisms contributing to age-related hearing loss. Our findings provide additional evidence that early- and later-onset SNHL may involve partly distinct biological mechanisms [63, 64].

Sex differences in auditory function are well established: although both sexes develop normal hearing sensitivity, females generally exhibit larger otoacoustic emissions and higher-amplitude auditory brainstem responses in adulthood, whereas males have a higher prevalence, greater severity, and earlier onset of hearing loss [65–67]. Animal models and cochlear transcriptomic studies further demonstrate sex-related differences in hearing loss mechanisms and gene expression [68–70]. Consistent with these observations, our sex-stratified analyses revealed sex differences in the effect sizes at various loci: we observed significantly larger genetic effect sizes in females for SNHL lead variants in or near *ILDR1*, *ARHGEF28*, *HLA*, *SYNJ2*, *MYO7A*, *KLC1*, and *TRIOBP*, whereas only the *POU4F1* locus showed a significantly larger effect in males. Although hearing loss is more common and severe in males, larger genetic effect sizes in females may reflect differences in environmental exposures and hormonal protection between sexes. Males are more frequently exposed to environmental risk factors such as occupational noise, as they are more likely to work in industries with high noise exposure [71, 72], which may dilute the relative contribution of genetic susceptibility. In contrast, estrogen-mediated protection of cochlear structures may increase the liability threshold for hearing loss in females [70], potentially amplifying the observable effects of genetic susceptibility among women who develop the condition. Consistent with a potential role of sex hormone signaling in hearing biology, we also identified an association near *ESR2*, which encodes estrogen receptor β. Although the effect size did not differ significantly between subgroups, the association appeared most robust among individuals diagnosed after the age of 55, potentially reflecting age-related changes in estrogen signaling. Notably, several loci showing stronger effects in females are involved in biological processes central to cochlear hair cell structure and function, including stereocilia integrity (*MYO7A*, *TRIOBP*), epithelial barrier and tight junction regulation (*ILDR1*), cytoskeletal and intracellular transport processes (*ARHGEF28*, *KLC1*), and synaptic vesicle trafficking (*SYNJ2*), suggesting that sex-dependent modulation of hair cell resilience may contribute to differential genetic effects. In contrast, the *POU4F1* locus, which showed a larger effect in males, encodes a transcription factor involved in the development and survival of sensory neurons, including spiral ganglion neurons in the auditory pathway [73]. Sex-specific immune responses may also contribute to these differences, particularly for the HLA locus, which has been implicated in immune-mediated traits and complex hearing phenotypes [14, 74, 75]. Overall, these findings support sex-dependent genetic effects in SNHL, consistent with sex-stratified results reported in a previous GWAS on hearing problems [76].

### Limitations and Conclusions

We have only employed computational methods and, thus, our findings would benefit from further functional analyses to identify truly causal genes. The eQTL colocalization analysis lacks sensory tissue-related data, such as cochlea-specific datasets, which would be relevant to this study. Additionally, the downstream analyses conducted in the analytical subgroups are not directly comparable to the findings from the full sample due to reduced statistical power for locus discovery and should therefore be interpreted with caution. Replicating these findings in diverse ethnic groups would enhance generalizability, as our sample is limited to European ancestry, particularly the distinct Finnish-Estonian gene pool.

In summary, this study advances the understanding of the genetic basis of SNHL by identifying multiple previously unreported loci and carrying out numerous downstream analyses. Interestingly, several SNHL-associated loci harbour genes involved in cochlear mechanotransduction and inner ear structures, highlighting biological pathways relevant to SNHL pathogenesis. Age- and sex-stratified approach provided additional insights into differences in the genetic contribution to SNHL across subgroups. Together, our findings deepen the understanding of the genetic risk factors of SNHL and provide a foundation for future studies investigating its biological mechanisms, novel therapies, and potential preventative strategies.

Gene, a plausible candidate the biological function of which is likely to explain the SNHL association; CHR:POS, chromosome, and position (genome build hg38); rsid, SNP markers identification number; EA, SNHL risk increasing allele; OA, other allele; *P*-value, Cohort, study population or subgroup in age- or sex-stratified analysis; OR (95CI), odds ratio and its 95% confidence interval, EAF, effect allele frequency. All = General population, O55 = those who were diagnosed at the age of 55 or after, Y55 = those who were diagnosed before the age of 55

## Supplementary Information

Below is the link to the electronic supplementary material.

ESM 1 (DOCX 4.46 MB)

ESM 2 (XLSX 4.46 MB)

## Data Availability

FinnGen summary statistics are available via Fingenious® (https://site.fingenious.fi/en/). Individual-level data are available to approved researchers through Finnish biobank application procedures. GTEx (https://gtexportal.org), gnomAD (https://gnomad.broadinstitute.org), eQTL Catalogue (https://www.ebi.ac.uk/eqtl/), and gEAR (https://umgear.org) data are publicly available. Custom GWAS summary statistics and experimental image data are available from the corresponding author upon reasonable request.

## Code Availability

We used open-source tools that have been referenced in the Methods section.

## Declarations

### Use of AI

No AI used.

## Abbreviations

SNHL: Sensorineural hearing loss
GWAS: Genome wide associated study
MAF: Minor allele frequency
EstBB: Estonian Biobank
HWE: Hardy–Weinberg equilibrium
MAC: Minor allele count
ARHL: Age-related hearing loss
EHR: Electronic health records
QC: Quality control
CHR: POS Chromosome and position (genome build hg38)
rsid: SNP marker identification number
EA: SNHL risk-increasing allele
OA: Other allele
OR (95CI): Odds ratio and its 95% confidence interval
EAF: Effect allele frequency
All: General population
O55: Those who were diagnosed at the age of 55 or after
Y55: Those who were diagnosed before the age of 55
