# Supplementary material for: Discovering Age- and Sex-Specific Genetic Risk Factors in Sensorineural Hearing Loss: Genome-Wide Evidence from Large-Scale Biobanks
Source: J Assoc Res Otolaryngol. 2026 May 26;27(4):631–46. doi: 10.1007/s10162-026-01054-y (PMC13504140; doi:10.1007/s10162-026-01054-y)
Supplement: Supplementary file 1 [file 10162_2026_1054_MOESM1_ESM.docx]

**Discovering age- and sex-specific genetic risk factors in sensorineural hearing loss: genome-wide evidence from large-scale biobanks**

Argyro Bizaki-Vallaskangas^1*^, Eeva Sliz^2^, Tuuli Lankinen^3^, Elmo Saarentaus^4^, Ville Salo^2^, Kristi Krebs^5^, Tytti Willberg^6^, Ilkka Kivekäs^1^, Joel Rämö^4^, Sanna Toppila-Salmi^7,8^, Aarno Dietz^7^, Vesa Hytönen^9^, Aarno Palotie^4,10,11^, Lili Milani^5^, Antti Mäkitie^12,13^, Johannes Kettunen^2^

^1 Faculty of Medicine and Health Technology, Department of Otolaryngology, University of Tampere, Tampere, Finland^

^2 Research Unit of Population Health, Biocenter Oulu, and Medical Research Center Oulu, University of Oulu and Oulu University Hospital, Oulu, Finland^

^3 Faculty of Medicine, University of Helsinki, Helsinki, Finland^

^4 Institute for Molecular Medicine Finland (FIMM), HiLIFE, University of Helsinki, Helsinki, Finland.^

^5 Estonian Genome Centre, Institute of Genomics, University of Tartu, Tartu, Estonia^

^6 Turku University Hospital, Department of Otolaryngology, Turku, Finland^

^7 Department of Otorhinolaryngology, University of Eastern Finland, Joensuu and Kuopio, Finland ,Wellbeing services county of Pohjois-Savo, Kuopio, Finland^

^8 Department of Allergology, Inflammation Center, Helsinki University Hospital and University of Helsinki, Helsinki, Finland^

^9 Department of Medicine and Health Technology, University of Tampere, Tampere, Finland and Fimlab Laboratories, Tampere, Finland^

^10 The Stanley Center for Psychiatric Research and Program in Medical and Population Genetics, The Broad Institute of MIT and Harvard, Cambridge, MA, USA.^

^11 Analytic and Translational Genetics Unit, Department of Medicine, Department of Neurology and Department of Psychiatry Massachusetts General Hospital, Boston, MA, USA.^

^12 Department of Otorhinolaryngology – Head and Neck Surgery, Helsinki University Hospital and University of Helsinki, Helsinki, Finland^

^13 Research Program in Systems Oncology, Faculty of Medicine, University of Helsinki, Helsinki, Finland^

*Corresponding author: Argyro Bizaki-Vallaskangas (arbizaki@gmail.com)

**Table of contents**

**Figure S1.** Lead variant effect estimates in every dataset

**Figure S2.** Previously unreported SNHL association on chromosome 13 in general population

**Figure S3.** Previously unreported SNHL association on chromosome 16 in general population

**Figure S4.** Previously unreported SNHL association on chromosome 20 in general population

**Figure S5.** Previously unreported SNHL association on chromosome 5 in cases diagnosed at the age of 55 or older

**Figure S6.** Previously unreported SNHL association on chromosome 10 in cases diagnosed at the age of 55 or older

**Figure S7.** Previously unreported SNHL association on chromosome 18 in cases diagnosed before the age of 55

**Figure S8.** Previously unreported SNHL association on chromosome 11 in females

**Figure S9.** Previously unreported SNHL association on chromosome 14 in females

**Figure S10.** Previously unreported SNHL association on chromosome 13 in males

**Figure S11.** Magma gene-set analysis in subgroups

**Figure S12.** Gene expression profiles for candidate genes in ear related cell types

**Figure S13.** eQTL colocalization results

**Table S1.** A list of FinnGen authors and their affiliations

**Table S2.** A list of Estonian Biobank Research Team members and their affiliations

**Table S3.** Lead variants of the SNHL associated loci identified in the genome-wide meta-analysis focusing general population

**Table S4.** The lead variants of the SNHL-associated loci associated in the genome-wide meta-analysis focusing cases diagnosed at the age of 55 or older

**Table S5.** The lead variants of the SNHL-associated loci associated in the genome-wide meta-analysis focusing cases diagnosed before the age of 55

**Table S6.** Lead variants of the SNHL associated loci identified in the genome-wide meta-analysis focusing female subgroup

**Table S7.** Lead variants of the SNHL associated loci identified in the genome-wide meta-analysis focusing male subgroup

**Tables S8 and S9 are provided in a separate excel file.**

## **Figure S1.** The effect estimates and 95% confidence intervals for SNHL-associated lead variants in each meta-analysis [black] and each cohort separately FinnGen [orange], Estonian Biobank [blue] are shown. Chromosome, position and effect allele were used as lead variant identifiers.

## **Figure S2.** Regional association plot of previously unreported SNHL association on chromosome 13 area of 39.5-40.5 MB, which was observed in meta-analysis focusing general population. Our results suggest that *COG6* (*component of oligomeric golgi complex 6*) is the gene most likely responsible for the association of the locus. Plot was generated by using Locuszoom <https://my.locuszoom.org/>. For LD estimations, the 1000G data-based LD reference panel with a European subpopulation was utilised.

## **Figure S3.** Regional association plot of previously unreported SNHL association on chromosome 16 area of 88.0-89.0 MB, which was observed in meta-analysis focusing general population. Our results suggest that *PIEZO1* *(piezo type mechanosensitive ion channel component 1*) is the gene most likely responsible for the association of the locus. Plot was generated by using Locuszoom <https://my.locuszoom.org/>. For LD estimations, the 1000G data-based LD reference panel with a European subpopulation was utilised.

## **Figure S4.** Regional association plot of previously unreported SNHL association on chromosome 20 area of 60.7-61.7 MB, which was observed in meta-analysis focusing general population. Our results suggest that *CDH4* (*cadherin 4*) is the gene most likely responsible for the association of the locus. Plot was generated by using Locuszoom <https://my.locuszoom.org/>. For LD estimations, the 1000G data-based LD reference panel with a European subpopulation was utilised.

## **Figure S5.** Regional association plot of previously unreported SNHL association on chromosome 5 area of 50.7-51.7 MB, which was observed in meta-analysis focusing cases diagnosed at the age of 55 or older. Our results suggest that *ISL1* (*ISL LIM homeobox 1*) is the gene most likely responsible for the association of the locus. Plot was generated by using Locuszoom <https://my.locuszoom.org/>. For LD estimations, the 1000G data-based LD reference panel with a European subpopulation was utilised.

## **Figure S6.** Regional association plot of previously unreported SNHL association on chromosome 10 area of 69.3-70.3 MB, which was observed in meta-analysis focusing cases diagnosed at the age of 55 or older. Our results suggest that *COL13A1* (*collagen type XIII alpha 1 chain*) is the gene most likely responsible for the association of the locus. Plot was generated by using Locuszoom <https://my.locuszoom.org/>. For LD estimations, the 1000G data-based LD reference panel with a European subpopulation was utilised.

## **Figure S7** Regional association plot of previously unreported SNHL association on chromosome 18 area of 39.4-40.4 MB, which was observed in meta-analysis focusing cases diagnosed before the age of 55. There was not any protein coding gene in this locus, so it was classified as *LINC01901* (*long intergenic non-protein coding RNA 1901*). Plot was generated by using Locuszoom <https://my.locuszoom.org/>. For LD estimations, the 1000G data-based LD reference panel with a European subpopulation was utilised.

## **Figure S8.** Regional association plot of previously unreported SNHL association on chromosome 11 area of 76.7-77.7 MB, which was observed in meta-analysis focusing female subgroup. Our results suggest that *MYO7A* (*myosin VIIA*) is the gene most likely responsible for the association of the locus. Plot was generated by using Locuszoom <https://my.locuszoom.org/>. For LD estimations, the 1000G data-based LD reference panel with a European subpopulation was utilised.

## **Figure S9.** Regional association plot of previously unreported SNHL association on chromosome 14 area of 103.2-104.2 MB, which was observed in meta-analysis focusing female subgroup. Our results suggest that *KLC1* (*kinesin light chain 1*) is the gene most likely responsible for the association of the locus. Plot was generated by using Locuszoom <https://my.locuszoom.org/>. For LD estimations, the 1000G data-based LD reference panel with a European subpopulation was utilised.

## **Figure S10.** Regional association plot of previously unreported SNHL association on chromosome 13 area of 78.3-79.3 MB, which was observed in meta-analysis focusing male subgroup. Our results suggest that *POU4F1* ( *POU class 4 homeobox 1*) is the gene most likely responsible for the association of the locus. Plot was generated by using Locuszoom <https://my.locuszoom.org/>. For LD estimations, the 1000G data-based LD reference panel with a European subpopulation was utilised.

**Figure S11. MAGMA gene-set analysis.** We conducted MAGMA gene-set analysis using 34,494 curated gene sets and Gene Ontology (GO) terms from the MSigDB (Liberzon et al., 2011) (version 2023.3). The plot displays the 20 most significant gene sets, with two highlighted in orange, indicating significance following false discovery rate (FDR) correction. All, full sample; o55, older than 55; y55, younger than 55.

**Figure S12.** Gene expression profiles for 24 candidate genes in ear related cell types. Gene expression profiles were retrieved from the eEAR^1^, using the Human inner ear atlas datasets^2^. In total, information on the expression of candidate genes was obtained from 18 different cell types. In the plot, the size of the dot indicates how large a fraction of cells of that cell type express the gene, and the intensity of the color indicates the log2 mean expression of the gene.

**Figure S13.** Gene expression and observed SNHL associations were colocalized using the "coloc" R-library function ‘coloc.abf’^3^. For every lead variant, colocalization was evaluated for every gene within a 1MB window. From the GTExportal (<https://www.gtexportal.org/home/datasets>), we downloaded variant-gene expression associations (GTEx v8, accessed on 19/05/2022) for the analysis. SNHL-relevant tissues were used in the analysis, including whole blood, brain (cerebellum, cortex, and hippocampus), adipose (subcutaneous, visceral), and tibial nerve. We utilised ≥ 0.8 posterior probabilities for the shared causal variant (PP4) as a limit for significant results^4^.

**Table S1.** A list of FinnGen authors and their affiliations

| Full Name | Affiliation | Role 1 | Role 2 |
| --- | --- | --- | --- |
| Aarno Palotie | Institute for Molecular Medicine Finland (FIMM), HiLIFE, University of Helsinki, Helsinki, Finland; Broad Institute of MIT and Harvard; Massachusetts General Hospital | **Steering Committee** | **Steering Committee** |
| Mark Daly | Institute for Molecular Medicine Finland (FIMM), HiLIFE, University of Helsinki, Helsinki, Finland; Broad Institute of MIT and Harvard; Massachusetts General Hospital | **Steering Committee** | **Steering Committee** |
| Bridget Riley-Gills | Abbvie, Chicago, IL, United States | **Steering Committee** | **Pharmaceutical companies** |
| Howard Jacob | Abbvie, Chicago, IL, United States | **Steering Committee** | **Pharmaceutical companies** |
| Dirk Paul | Astra Zeneca, Cambridge, United Kingdom | **Steering Committee** | **Pharmaceutical companies** |
| Slavé Petrovski | Astra Zeneca, Cambridge, United Kingdom | **Steering Committee** | **Pharmaceutical companies** |
| Heiko Runz | Biogen, Cambridge, MA, United States | **Steering Committee** | **Pharmaceutical companies** |
| Sally John | Biogen, Cambridge, MA, United States | **Steering Committee** | **Pharmaceutical companies** |
| George Okafo | Boehringer Ingelheim, Ingelheim am Rhein, Germany | **Steering Committee** | **Pharmaceutical companies** |
| Nathan Lawless | Boehringer Ingelheim, Ingelheim am Rhein, Germany | **Steering Committee** | **Pharmaceutical companies** |
| Heli Salminen-Mankonen | Boehringer Ingelheim, Ingelheim am Rhein, Germany | **Steering Committee** | **Pharmaceutical companies** |
| Robert Plenge | Bristol Myers Squibb, New York, NY, United States | **Steering Committee** | **Pharmaceutical companies** |
| Joseph Maranville | Bristol Myers Squibb, New York, NY, United States | **Steering Committee** | **Pharmaceutical companies** |
| Mark McCarthy | Genentech, San Francisco, CA, United States | **Steering Committee** | **Pharmaceutical companies** |
| Margaret G. Ehm | GlaxoSmithKline, Collegeville, PA, United States | **Steering Committee** | **Pharmaceutical companies** |
| Kirsi Auro | GlaxoSmithKline, Espoo, Finland | **Steering Committee** | **Pharmaceutical companies** |
| Simonne Longerich | Merck, Kenilworth, NJ, United States | **Steering Committee** | **Pharmaceutical companies** |
| Anders Mälarstig | Pfizer, New York, NY, United States | **Steering Committee** | **Pharmaceutical companies** |
| Katherine Klinger | Translational Sciences, Sanofi R&D, Framingham, MA, USA | **Steering Committee** | **Pharmaceutical companies** |
| Clement Chatelain | Translational Sciences, Sanofi R&D, Framingham, MA, USA | **Steering Committee** | **Pharmaceutical companies** |
| Matthias Gossel | Translational Sciences, Sanofi R&D, Framingham, MA, USA | **Steering Committee** | **Pharmaceutical companies** |
| Karol Estrada | Maze Therapeutics, San Francisco, CA, United States | **Steering Committee** | **Pharmaceutical companies** |
| Robert Graham | Maze Therapeutics, San Francisco, CA, United States | **Steering Committee** | **Pharmaceutical companies** |
| Robert Yang | Janssen Biotech, Beerse, Belgium | **Steering Committee** | **Pharmaceutical companies** |
| Chris O´Donnell | Novartis Institutes for BioMedical Research, Cambridge, MA, United States | **Steering Committee** | **Pharmaceutical companies** |
| Tomi P. Mäkelä | HiLIFE, University of Helsinki, Finland, Finland | **Steering Committee** | **University of Helsinki & Biobanks** |
| Jaakko Kaprio | Institute for Molecular Medicine Finland (FIMM), HiLIFE, University of Helsinki, Helsinki, Finland | **Steering Committee** | **University of Helsinki & Biobanks** |
| Petri Virolainen | Auria Biobank / University of Turku / Hospital District of Southwest Finland, Turku, Finland | **Steering Committee** | **University of Helsinki & Biobanks** |
| Antti Hakanen | Auria Biobank / University of Turku / Hospital District of Southwest Finland, Turku, Finland | **Steering Committee** | **University of Helsinki & Biobanks** |
| Terhi Kilpi | THL Biobank / Finnish Institute for Health and Welfare (THL), Helsinki, Finland | **Steering Committee** | **University of Helsinki & Biobanks** |
| Markus Perola | THL Biobank / Finnish Institute for Health and Welfare (THL), Helsinki, Finland | **Steering Committee** | **University of Helsinki & Biobanks** |
| Jukka Partanen | Finnish Red Cross Blood Service / Finnish Hematology Registry and Clinical Biobank, Helsinki, Finland | **Steering Committee** | **University of Helsinki & Biobanks** |
| Anne Pitkäranta | Helsinki Biobank / Helsinki University and Hospital District of Helsinki and Uusimaa, Helsinki | **Steering Committee** | **University of Helsinki & Biobanks** |
| Taneli Raivio | Helsinki Biobank / Helsinki University and Hospital District of Helsinki and Uusimaa, Helsinki | **Steering Committee** | **University of Helsinki & Biobanks** |
| Jani Tikkanen | Northern Finland Biobank Borealis / University of Oulu / Northern Ostrobothnia Hospital District, Oulu, Finland | **Steering Committee** | **University of Helsinki & Biobanks** |
| Raisa Serpi | Northern Finland Biobank Borealis / University of Oulu / Northern Ostrobothnia Hospital District, Oulu, Finland | **Steering Committee** | **University of Helsinki & Biobanks** |
| Tarja Laitinen | Finnish Clinical Biobank Tampere / University of Tampere / Pirkanmaa Hospital District, Tampere, Finland | **Steering Committee** | **University of Helsinki & Biobanks** |
| Veli-Matti Kosma | Biobank of Eastern Finland / University of Eastern Finland / Northern Savo Hospital District, Kuopio, Finland | **Steering Committee** | **University of Helsinki & Biobanks** |
| Jari Laukkanen | Central Finland Biobank / University of Jyväskylä / Central Finland Health Care District, Jyväskylä, Finland | **Steering Committee** | **University of Helsinki & Biobanks** |
| Marco Hautalahti | FINBB - Finnish biobank cooperative | **Steering Committee** | **University of Helsinki & Biobanks** |
| Outi Tuovila | Business Finland, Helsinki, Finland | **Steering Committee** | **Other Experts/ Non-Voting Members** |
| Raimo Pakkanen | Business Finland, Helsinki, Finland | **Steering Committee** | **Other Experts/ Non-Voting Members** |
| Jeffrey Waring | Abbvie, Chicago, IL, United States | **Scientific Committee** | **Pharmaceutical companies** |
| Bridget Riley-Gillis | Abbvie, Chicago, IL, United States | **Scientific Committee** | **Pharmaceutical companies** |
| Fedik Rahimov | Abbvie, Chicago, IL, United States | **Scientific Committee** | **Pharmaceutical companies** |
| Ioanna Tachmazidou | Astra Zeneca, Cambridge, United Kingdom | **Scientific Committee** | **Pharmaceutical companies** |
| Chia-Yen Chen | Biogen, Cambridge, MA, United States | **Scientific Committee** | **Pharmaceutical companies** |
| Heiko Runz | Biogen, Cambridge, MA, United States | **Scientific Committee** | **Pharmaceutical companies** |
| Zhihao Ding | Boehringer Ingelheim, Ingelheim am Rhein, Germany | **Scientific Committee** | **Pharmaceutical companies** |
| Marc Jung | Boehringer Ingelheim, Ingelheim am Rhein, Germany | **Scientific Committee** | **Pharmaceutical companies** |
| Shameek Biswas | Bristol Myers Squibb, New York, NY, United States | **Scientific Committee** | **Pharmaceutical companies** |
| Rion Pendergrass | Genentech, San Francisco, CA, United States | **Scientific Committee** | **Pharmaceutical companies** |
| Margaret G. Ehm | GlaxoSmithKline, Collegeville, PA, United States | **Scientific Committee** | **Pharmaceutical companies** |
| David Pulford | GlaxoSmithKline, Stevenage, United Kingdom | **Scientific Committee** | **Pharmaceutical companies** |
| Neha Raghavan | Merck, Kenilworth, NJ, United States | **Scientific Committee** | **Pharmaceutical companies** |
| Adriana Huertas-Vazquez | Merck, Kenilworth, NJ, United States | **Scientific Committee** | **Pharmaceutical companies** |
| Jae-Hoon Sul | Merck, Kenilworth, NJ, United States | **Scientific Committee** | **Pharmaceutical companies** |
| Anders Mälarstig | Pfizer, New York, NY, United States | **Scientific Committee** | **Pharmaceutical companies** |
| Xinli Hu | Pfizer, New York, NY, United States | **Scientific Committee** | **Pharmaceutical companies** |
| Åsa Hedman | Pfizer, New York, NY, United States | **Scientific Committee** | **Pharmaceutical companies** |
| Katherine Klinger | Translational Sciences, Sanofi R&D, Framingham, MA, USA | **Scientific Committee** | **Pharmaceutical companies** |
| Robert Graham | Maze Therapeutics, San Francisco, CA, United States | **Scientific Committee** | **Pharmaceutical companies** |
| Manuel Rivas | Maze Therapeutics, San Francisco, CA, United States | **Scientific Committee** | **Pharmaceutical companies** |
| Dawn Waterworth | Janssen Research & Development, LLC, Spring House, PA, United States | **Scientific Committee** | **Pharmaceutical companies** |
| Nicole Renaud | Novartis Institutes for BioMedical Research, Cambridge, MA, United States | **Scientific Committee** | **Pharmaceutical companies** |
| Ma´en Obeidat | Novartis Institutes for BioMedical Research, Cambridge, MA, United States | **Scientific Committee** | **Pharmaceutical companies** |
| Samuli Ripatti | Institute for Molecular Medicine Finland (FIMM), HiLIFE, University of Helsinki, Helsinki, Finland | **Scientific Committee** | **University of Helsinki & Biobanks** |
| Johanna Schleutker | Auria Biobank / Univ. of Turku / Hospital District of Southwest Finland, Turku, Finland | **Scientific Committee** | **University of Helsinki & Biobanks** |
| Markus Perola | THL Biobank / Finnish Institute for Health and Welfare (THL), Helsinki, Finland | **Scientific Committee** | **University of Helsinki & Biobanks** |
| Mikko Arvas | Finnish Red Cross Blood Service / Finnish Hematology Registry and Clinical Biobank, Helsinki, Finland | **Scientific Committee** | **University of Helsinki & Biobanks** |
| Olli Carpén | Helsinki Biobank / Helsinki University and Hospital District of Helsinki and Uusimaa, Helsinki | **Scientific Committee** | **University of Helsinki & Biobanks** |
| Reetta Hinttala | Northern Finland Biobank Borealis / University of Oulu / Northern Ostrobothnia Hospital District, Oulu, Finland | **Scientific Committee** | **University of Helsinki & Biobanks** |
| Johannes Kettunen | Northern Finland Biobank Borealis / University of Oulu / Northern Ostrobothnia Hospital District, Oulu, Finland | **Scientific Committee** | **University of Helsinki & Biobanks** |
| Arto Mannermaa | Biobank of Eastern Finland / University of Eastern Finland / Northern Savo Hospital District, Kuopio, Finland | **Scientific Committee** | **University of Helsinki & Biobanks** |
| Katriina Aalto-Setälä | Faculty of Medicine and Health Technology, Tampere University, Tampere, Finland | **Scientific Committee** | **University of Helsinki & Biobanks** |
| Mika Kähönen | Finnish Clinical Biobank Tampere / University of Tampere / Pirkanmaa Hospital District, Tampere, Finland | **Scientific Committee** | **University of Helsinki & Biobanks** |
| Jari Laukkanen | Central Finland Biobank / University of Jyväskylä / Central Finland Health Care District, Jyväskylä, Finland | **Scientific Committee** | **University of Helsinki & Biobanks** |
| Johanna Mäkelä | FINBB - Finnish biobank cooperative | **Scientific Committee** | **University of Helsinki & Biobanks** |
| Reetta Kälviäinen | Northern Savo Hospital District, Kuopio, Finland | **Clinical Groups** | **Neurology Group** |
| Valtteri Julkunen | Northern Savo Hospital District, Kuopio, Finland | **Clinical Groups** | **Neurology Group** |
| Hilkka Soininen | Northern Savo Hospital District, Kuopio, Finland | **Clinical Groups** | **Neurology Group** |
| Anne Remes | Northern Ostrobothnia Hospital District, Oulu, Finland | **Clinical Groups** | **Neurology Group** |
| Mikko Hiltunen | University of Eastern Finland, Kuopio, Finland | **Clinical Groups** | **Neurology Group** |
| Jukka Peltola | Pirkanmaa Hospital District, Tampere, Finland | **Clinical Groups** | **Neurology Group** |
| Minna Raivio | Hospital District of Helsinki and Uusimaa, Helsinki, Finland | **Clinical Groups** | **Neurology Group** |
| Pentti Tienari | Hospital District of Helsinki and Uusimaa, Helsinki, Finland | **Clinical Groups** | **Neurology Group** |
| Juha Rinne | Hospital District of Southwest Finland, Turku, Finland | **Clinical Groups** | **Neurology Group** |
| Roosa Kallionpää | Hospital District of Southwest Finland, Turku, Finland | **Clinical Groups** | **Neurology Group** |
| Juulia Partanen | Institute for Molecular Medicine Finland, HiLIFE, University of Helsinki, Finland | **Clinical Groups** | **Neurology Group** |
| Ali Abbasi | Abbvie, Chicago, IL, United States | **Clinical Groups** | **Neurology Group** |
| Adam Ziemann | Abbvie, Chicago, IL, United States | **Clinical Groups** | **Neurology Group** |
| Nizar Smaoui | Abbvie, Chicago, IL, United States | **Clinical Groups** | **Neurology Group** |
| Anne Lehtonen | Abbvie, Chicago, IL, United States | **Clinical Groups** | **Neurology Group** |
| Susan Eaton | Biogen, Cambridge, MA, United States | **Clinical Groups** | **Neurology Group** |
| Heiko Runz | Biogen, Cambridge, MA, United States | **Clinical Groups** | **Neurology Group** |
| Sanni Lahdenperä | Biogen, Cambridge, MA, United States | **Clinical Groups** | **Neurology Group** |
| Shameek Biswas | Bristol Myers Squibb, New York, NY, United States | **Clinical Groups** | **Neurology Group** |
| Natalie Bowers | Genentech, San Francisco, CA, United States | **Clinical Groups** | **Neurology Group** |
| Edmond Teng | Genentech, San Francisco, CA, United States | **Clinical Groups** | **Neurology Group** |
| Rion Pendergrass | Genentech, San Francisco, CA, United States | **Clinical Groups** | **Neurology Group** |
| Fanli Xu | GlaxoSmithKline, Brentford, United Kingdom | **Clinical Groups** | **Neurology Group** |
| David Pulford | GlaxoSmithKline, Stevenage, United Kingdom | **Clinical Groups** | **Neurology Group** |
| Kirsi Auro | GlaxoSmithKline, Espoo, Finland | **Clinical Groups** | **Neurology Group** |
| Laura Addis | GlaxoSmithKline, Brentford, United Kingdom | **Clinical Groups** | **Neurology Group** |
| John Eicher | GlaxoSmithKline, Brentford, United Kingdom | **Clinical Groups** | **Neurology Group** |
| Qingqin S Li | Janssen Research & Development, LLC, Titusville, NJ 08560, United States | **Clinical Groups** | **Neurology Group** |
| Karen He | Janssen Research & Development, LLC, Spring House, PA, United States | **Clinical Groups** | **Neurology Group** |
| Ekaterina Khramtsova | Janssen Research & Development, LLC, Spring House, PA, United States | **Clinical Groups** | **Neurology Group** |
| Neha Raghavan | Merck, Kenilworth, NJ, United States | **Clinical Groups** | **Neurology Group** |
| Martti Färkkilä | Hospital District of Helsinki and Uusimaa, Helsinki, Finland | **Clinical Groups** | **Gastroenterology Group** |
| Jukka Koskela | Hospital District of Helsinki and Uusimaa, Helsinki, Finland | **Clinical Groups** | **Gastroenterology Group** |
| Sampsa Pikkarainen | Hospital District of Helsinki and Uusimaa, Helsinki, Finland | **Clinical Groups** | **Gastroenterology Group** |
| Airi Jussila | Pirkanmaa Hospital District, Tampere, Finland | **Clinical Groups** | **Gastroenterology Group** |
| Katri Kaukinen | Pirkanmaa Hospital District, Tampere, Finland | **Clinical Groups** | **Gastroenterology Group** |
| Timo Blomster | Northern Ostrobothnia Hospital District, Oulu, Finland | **Clinical Groups** | **Gastroenterology Group** |
| Mikko Kiviniemi | Northern Savo Hospital District, Kuopio, Finland | **Clinical Groups** | **Gastroenterology Group** |
| Markku Voutilainen | Hospital District of Southwest Finland, Turku, Finland | **Clinical Groups** | **Gastroenterology Group** |
| Mark Daly | Institute for Molecular Medicine, Finland (FIMM), HiLIFE, University of Helsinki, Helsinki, Finland; Broad Institute of MIT and Harvard; Massachusetts General Hospital | **Clinical Groups** | **Gastroenterology Group** |
| Ali Abbasi | Abbvie, Chicago, IL, United States | **Clinical Groups** | **Gastroenterology Group** |
| Jeffrey Waring | Abbvie, Chicago, IL, United States | **Clinical Groups** | **Gastroenterology Group** |
| Nizar Smaoui | Abbvie, Chicago, IL, United States | **Clinical Groups** | **Gastroenterology Group** |
| Fedik Rahimov | Abbvie, Chicago, IL, United States | **Clinical Groups** | **Gastroenterology Group** |
| Anne Lehtonen | Abbvie, Chicago, IL, United States | **Clinical Groups** | **Gastroenterology Group** |
| Tim Lu | Genentech, San Francisco, CA, United States | **Clinical Groups** | **Gastroenterology Group** |
| Natalie Bowers | Genentech, San Francisco, CA, United States | **Clinical Groups** | **Gastroenterology Group** |
| Rion Pendergrass | Genentech, San Francisco, CA, United States | **Clinical Groups** | **Gastroenterology Group** |
| Linda McCarthy | GlaxoSmithKline, Brentford, United Kingdom | **Clinical Groups** | **Gastroenterology Group** |
| Amy Hart | Janssen Research & Development, LLC, Spring House, PA, United States | **Clinical Groups** | **Gastroenterology Group** |
| Meijian Guan | Janssen Research & Development, LLC, Spring House, PA, United States | **Clinical Groups** | **Gastroenterology Group** |
| Jason Miller | Merck, Kenilworth, NJ, United States | **Clinical Groups** | **Gastroenterology Group** |
| Kirsi Kalpala | Pfizer, New York, NY, United States | **Clinical Groups** | **Gastroenterology Group** |
| Melissa Miller | Pfizer, New York, NY, United States | **Clinical Groups** | **Gastroenterology Group** |
| Xinli Hu | Pfizer, New York, NY, United States | **Clinical Groups** | **Gastroenterology Group** |
| Kari Eklund | Hospital District of Helsinki and Uusimaa, Helsinki, Finland | **Clinical Groups** | **Rheumatology Group** |
| Antti Palomäki | Hospital District of Southwest Finland, Turku, Finland | **Clinical Groups** | **Rheumatology Group** |
| Pia Isomäki | Pirkanmaa Hospital District, Tampere, Finland | **Clinical Groups** | **Rheumatology Group** |
| Laura Pirilä | Hospital District of Southwest Finland, Turku, Finland | **Clinical Groups** | **Rheumatology Group** |
| Oili Kaipiainen-Seppänen | Northern Savo Hospital District, Kuopio, Finland | **Clinical Groups** | **Rheumatology Group** |
| Johanna Huhtakangas | Northern Ostrobothnia Hospital District, Oulu, Finland | **Clinical Groups** | **Rheumatology Group** |
| Nina Mars | Institute for Molecular Medicine Finland (FIMM), HiLIFE, University of Helsinki, Helsinki, Finland | **Clinical Groups** | **Rheumatology Group** |
| Ali Abbasi | Abbvie, Chicago, IL, United States | **Clinical Groups** | **Rheumatology Group** |
| Jeffrey Waring | Abbvie, Chicago, IL, United States | **Clinical Groups** | **Rheumatology Group** |
| Fedik Rahimov | Abbvie, Chicago, IL, United States | **Clinical Groups** | **Rheumatology Group** |
| Apinya Lertratanakul | Abbvie, Chicago, IL, United States | **Clinical Groups** | **Rheumatology Group** |
| Nizar Smaoui | Abbvie, Chicago, IL, United States | **Clinical Groups** | **Rheumatology Group** |
| Anne Lehtonen | Abbvie, Chicago, IL, United States | **Clinical Groups** | **Rheumatology Group** |
| Coralie Viollet | AstraZeneca, Cambridge, United Kingdom | **Clinical Groups** | **Rheumatology Group** |
| Marla Hochfeld | Bristol Myers Squibb, New York, NY, United States | **Clinical Groups** | **Rheumatology Group** |
| Natalie Bowers | Genentech, San Francisco, CA, United States | **Clinical Groups** | **Rheumatology Group** |
| Rion Pendergrass | Genentech, San Francisco, CA, United States | **Clinical Groups** | **Rheumatology Group** |
| Jorge Esparza Gordillo | GlaxoSmithKline, Brentford, United Kingdom | **Clinical Groups** | **Rheumatology Group** |
| Kirsi Auro | GlaxoSmithKline, Espoo, Finland | **Clinical Groups** | **Rheumatology Group** |
| Dawn Waterworth | Janssen Research & Development, LLC, Spring House, PA, United States | **Clinical Groups** | **Rheumatology Group** |
| Fabiana Farias | Merck, Kenilworth, NJ, United States | **Clinical Groups** | **Rheumatology Group** |
| Kirsi Kalpala | Pfizer, New York, NY, United States | **Clinical Groups** | **Rheumatology Group** |
| Nan Bing | Pfizer, New York, NY, United States | **Clinical Groups** | **Rheumatology Group** |
| Xinli Hu | Pfizer, New York, NY, United States | **Clinical Groups** | **Rheumatology Group** |
| Tarja Laitinen | Pirkanmaa Hospital District, Tampere, Finland | **Clinical Groups** | **Pulmonology Group** |
| Margit Pelkonen | Northern Savo Hospital District, Kuopio, Finland | **Clinical Groups** | **Pulmonology Group** |
| Paula Kauppi | Hospital District of Helsinki and Uusimaa, Helsinki, Finland | **Clinical Groups** | **Pulmonology Group** |
| Hannu Kankaanranta | University of Gothenburg, Gothenburg, Sweden/ Seinäjoki Central Hospital, Seinäjoki, Finland/ Tampere University, Tampere, Finland | **Clinical Groups** | **Pulmonology Group** |
| Terttu Harju | Northern Ostrobothnia Hospital District, Oulu, Finland | **Clinical Groups** | **Pulmonology Group** |
| Riitta Lahesmaa | Hospital District of Southwest Finland, Turku, Finland | **Clinical Groups** | **Pulmonology Group** |
| Nizar Smaoui | Abbvie, Chicago, IL, United States | **Clinical Groups** | **Pulmonology Group** |
| Coralie Viollet | AstraZeneca, Cambridge, United Kingdom | **Clinical Groups** | **Pulmonology Group** |
| Susan Eaton | Biogen, Cambridge, MA, United States | **Clinical Groups** | **Pulmonology Group** |
| Hubert Chen | Genentech, San Francisco, CA, United States | **Clinical Groups** | **Pulmonology Group** |
| Rion Pendergrass | Genentech, San Francisco, CA, United States | **Clinical Groups** | **Pulmonology Group** |
| Natalie Bowers | Genentech, San Francisco, CA, United States | **Clinical Groups** | **Pulmonology Group** |
| Joanna Betts | GlaxoSmithKline, Brentford, United Kingdom | **Clinical Groups** | **Pulmonology Group** |
| Kirsi Auro | GlaxoSmithKline, Espoo, Finland | **Clinical Groups** | **Pulmonology Group** |
| Rajashree Mishra | GlaxoSmithKline, Brentford, United Kingdom | **Clinical Groups** | **Pulmonology Group** |
| Majd Mouded | Novartis, Basel, Switzerland | **Clinical Groups** | **Pulmonology Group** |
| Debby Ngo | Novartis, Basel, Switzerland | **Clinical Groups** | **Pulmonology Group** |
| Teemu Niiranen | Finnish Institute for Health and Welfare (THL), Helsinki, Finland | **Clinical Groups** | **Cardiometabolic Diseases Group** |
| Felix Vaura | Finnish Institute for Health and Welfare (THL), Helsinki, Finland | **Clinical Groups** | **Cardiometabolic Diseases Group** |
| Veikko Salomaa | Finnish Institute for Health and Welfare (THL), Helsinki, Finland | **Clinical Groups** | **Cardiometabolic Diseases Group** |
| Kaj Metsärinne | Hospital District of Southwest Finland, Turku, Finland | **Clinical Groups** | **Cardiometabolic Diseases Group** |
| Jenni Aittokallio | Hospital District of Southwest Finland, Turku, Finland | **Clinical Groups** | **Cardiometabolic Diseases Group** |
| Mika Kähönen | Pirkanmaa Hospital District, Tampere, Finland | **Clinical Groups** | **Cardiometabolic Diseases Group** |
| Jussi Hernesniemi | Pirkanmaa Hospital District, Tampere, Finland | **Clinical Groups** | **Cardiometabolic Diseases Group** |
| Daniel Gordin | Hospital District of Helsinki and Uusimaa, Helsinki, Finland | **Clinical Groups** | **Cardiometabolic Diseases Group** |
| Juha Sinisalo | Hospital District of Helsinki and Uusimaa, Helsinki, Finland | **Clinical Groups** | **Cardiometabolic Diseases Group** |
| Marja-Riitta Taskinen | Hospital District of Helsinki and Uusimaa, Helsinki, Finland | **Clinical Groups** | **Cardiometabolic Diseases Group** |
| Tiinamaija Tuomi | Hospital District of Helsinki and Uusimaa, Helsinki, Finland | **Clinical Groups** | **Cardiometabolic Diseases Group** |
| Timo Hiltunen | Hospital District of Helsinki and Uusimaa, Helsinki, Finland | **Clinical Groups** | **Cardiometabolic Diseases Group** |
| Jari Laukkanen | Central Finland Health Care District, Jyväskylä, Finland | **Clinical Groups** | **Cardiometabolic Diseases Group** |
| Amanda Elliott | Institute for Molecular Medicine Finland (FIMM), HiLIFE, University of Helsinki, Helsinki, Finland; Broad Institute, Cambridge, MA, USA and Massachusetts General Hospital, Boston, MA, USA | **Clinical Groups** | **Cardiometabolic Diseases Group** |
| Mary Pat Reeve | Institute for Molecular Medicine Finland (FIMM), HiLIFE, University of Helsinki, Helsinki, Finland | **Clinical Groups** | **Cardiometabolic Diseases Group** |
| Sanni Ruotsalainen | Institute for Molecular Medicine Finland (FIMM), HiLIFE, University of Helsinki, Helsinki, Finland | **Clinical Groups** | **Cardiometabolic Diseases Group** |
| Dirk Paul | Astra Zeneca, Cambridge, United Kingdom | **Clinical Groups** | **Cardiometabolic Diseases Group** |
| Natalie Bowers | Genentech, San Francisco, CA, United States | **Clinical Groups** | **Cardiometabolic Diseases Group** |
| Rion Pendergrass | Genentech, San Francisco, CA, United States | **Clinical Groups** | **Cardiometabolic Diseases Group** |
| Audrey Chu | GlaxoSmithKline, Brentford, United Kingdom | **Clinical Groups** | **Cardiometabolic Diseases Group** |
| Kirsi Auro | GlaxoSmithKline, Espoo, Finland | **Clinical Groups** | **Cardiometabolic Diseases Group** |
| Dermot Reilly | Janssen Research & Development, LLC, Boston, MA, United States | **Clinical Groups** | **Cardiometabolic Diseases Group** |
| Mike Mendelson | Novartis, Boston, MA, United States | **Clinical Groups** | **Cardiometabolic Diseases Group** |
| Jaakko Parkkinen | Pfizer, New York, NY, United States | **Clinical Groups** | **Cardiometabolic Diseases Group** |
| Melissa Miller | Pfizer, New York, NY, United States | **Clinical Groups** | **Cardiometabolic Diseases Group** |
| Tuomo Meretoja | Hospital District of Helsinki and Uusimaa, Helsinki, Finland | **Clinical Groups** | **Oncology Group** |
| Heikki Joensuu | Hospital District of Helsinki and Uusimaa, Helsinki, Finland | **Clinical Groups** | **Oncology Group** |
| Olli Carpén | Hospital District of Helsinki and Uusimaa, Helsinki, Finland | **Clinical Groups** | **Oncology Group** |
| Johanna Mattson | Hospital District of Helsinki and Uusimaa, Helsinki, Finland | **Clinical Groups** | **Oncology Group** |
| Eveliina Salminen | Hospital District of Helsinki and Uusimaa, Helsinki, Finland | **Clinical Groups** | **Oncology Group** |
| Annika Auranen | Pirkanmaa Hospital District , Tampere, Finland | **Clinical Groups** | **Oncology Group** |
| Peeter Karihtala | Northern Ostrobothnia Hospital District, Oulu, Finland | **Clinical Groups** | **Oncology Group** |
| Päivi Auvinen | Northern Savo Hospital District, Kuopio, Finland | **Clinical Groups** | **Oncology Group** |
| Klaus Elenius | Hospital District of Southwest Finland, Turku, Finland | **Clinical Groups** | **Oncology Group** |
| Johanna Schleutker | Hospital District of Southwest Finland, Turku, Finland | **Clinical Groups** | **Oncology Group** |
| Esa Pitkänen | Institute for Molecular Medicine Finland (FIMM), HiLIFE, University of Helsinki, Helsinki, Finland | **Clinical Groups** | **Oncology Group** |
| Nina Mars | Institute for Molecular Medicine Finland (FIMM), HiLIFE, University of Helsinki, Helsinki, Finland | **Clinical Groups** | **Oncology Group** |
| Mark Daly | Institute for Molecular Medicine Finland (FIMM), HiLIFE, University of Helsinki, Helsinki, Finland; Broad Institute of MIT and Harvard; Massachusetts General Hospital | **Clinical Groups** | **Oncology Group** |
| Relja Popovic | Abbvie, Chicago, IL, United States | **Clinical Groups** | **Oncology Group** |
| Jeffrey Waring | Abbvie, Chicago, IL, United States | **Clinical Groups** | **Oncology Group** |
| Bridget Riley-Gillis | Abbvie, Chicago, IL, United States | **Clinical Groups** | **Oncology Group** |
| Anne Lehtonen | Abbvie, Chicago, IL, United States | **Clinical Groups** | **Oncology Group** |
| Margarete Fabre | AstraZeneca, Cambridge, United Kingdom | **Clinical Groups** | **Oncology Group** |
| Jennifer Schutzman | Genentech, San Francisco, CA, United States | **Clinical Groups** | **Oncology Group** |
| Natalie Bowers | Genentech, San Francisco, CA, United States | **Clinical Groups** | **Oncology Group** |
| Rion Pendergrass | Genentech, San Francisco, CA, United States | **Clinical Groups** | **Oncology Group** |
| Diptee Kulkarni | GlaxoSmithKline, Brentford, United Kingdom | **Clinical Groups** | **Oncology Group** |
| Kirsi Auro | GlaxoSmithKline, Espoo, Finland | **Clinical Groups** | **Oncology Group** |
| Alessandro Porello | Janssen Research & Development, LLC, Spring House, PA, United States | **Clinical Groups** | **Oncology Group** |
| Andrey Loboda | Merck, Kenilworth, NJ, United States | **Clinical Groups** | **Oncology Group** |
| Heli Lehtonen | Pfizer, New York, NY, United States | **Clinical Groups** | **Oncology Group** |
| Stefan McDonough | Pfizer, New York, NY, United States | **Clinical Groups** | **Oncology Group** |
| Sauli Vuoti | Janssen-Cilag Oy, Espoo, Finland | **Clinical Groups** | **Oncology Group** |
| Kai Kaarniranta | Northern Savo Hospital District, Kuopio, Finland; Department of Molecular Genetics, University of Lodz, Lodz, Poland | **Clinical Groups** | **Opthalmology Group** |
| Joni A Turunen | Helsinki University Hospital and University of Helsinki, Helsinki, Finland; Eye Genetics Group, Folkhälsan Research Center, Helsinki, Finland | **Clinical Groups** | **Opthalmology Group** |
| Terhi Ollila | Hospital District of Helsinki and Uusimaa, Helsinki, Finland | **Clinical Groups** | **Opthalmology Group** |
| Hannu Uusitalo | Pirkanmaa Hospital District, Tampere, Finland | **Clinical Groups** | **Opthalmology Group** |
| Juha Karjalainen | Institute for Molecular Medicine Finland (FIMM), HiLIFE, University of Helsinki, Helsinki, Finland | **Clinical Groups** | **Opthalmology Group** |
| Esa Pitkänen | Institute for Molecular Medicine Finland (FIMM), HiLIFE, University of Helsinki, Helsinki, Finland | **Clinical Groups** | **Opthalmology Group** |
| Mengzhen Liu | Abbvie, Chicago, IL, United States | **Clinical Groups** | **Opthalmology Group** |
| Heiko Runz | Biogen, Cambridge, MA, United States | **Clinical Groups** | **Opthalmology Group** |
| Stephanie Loomis | Biogen, Cambridge, MA, United States | **Clinical Groups** | **Opthalmology Group** |
| Erich Strauss | Genentech, San Francisco, CA, United States | **Clinical Groups** | **Opthalmology Group** |
| Natalie Bowers | Genentech, San Francisco, CA, United States | **Clinical Groups** | **Opthalmology Group** |
| Hao Chen | Genentech, San Francisco, CA, United States | **Clinical Groups** | **Opthalmology Group** |
| Rion Pendergrass | Genentech, San Francisco, CA, United States | **Clinical Groups** | **Opthalmology Group** |
| Kaisa Tasanen | Northern Ostrobothnia Hospital District, Oulu, Finland | **Clinical Groups** | **Dermatology Group** |
| Laura Huilaja | Northern Ostrobothnia Hospital District, Oulu, Finland | **Clinical Groups** | **Dermatology Group** |
| Katariina Hannula-Jouppi | Hospital District of Helsinki and Uusimaa, Helsinki, Finland | **Clinical Groups** | **Dermatology Group** |
| Teea Salmi | Pirkanmaa Hospital District, Tampere, Finland | **Clinical Groups** | **Dermatology Group** |
| Sirkku Peltonen | Hospital District of Southwest Finland, Turku, Finland | **Clinical Groups** | **Dermatology Group** |
| Leena Koulu | Hospital District of Southwest Finland, Turku, Finland | **Clinical Groups** | **Dermatology Group** |
| Nizar Smaoui | Abbvie, Chicago, IL, United States | **Clinical Groups** | **Dermatology Group** |
| Fedik Rahimov | Abbvie, Chicago, IL, United States | **Clinical Groups** | **Dermatology Group** |
| Anne Lehtonen | Abbvie, Chicago, IL, United States | **Clinical Groups** | **Dermatology Group** |
| David Choy | Genentech, San Francisco, CA, United States | **Clinical Groups** | **Dermatology Group** |
| Rion Pendergrass | Genentech, San Francisco, CA, United States | **Clinical Groups** | **Dermatology Group** |
| Dawn Waterworth | Janssen Research & Development, LLC, Spring House, PA, United States | **Clinical Groups** | **Dermatology Group** |
| Kirsi Kalpala | Pfizer, New York, NY, United States | **Clinical Groups** | **Dermatology Group** |
| Ying Wu | Pfizer, New York, NY, United States | **Clinical Groups** | **Dermatology Group** |
| Pirkko Pussinen | Hospital District of Helsinki and Uusimaa, Helsinki, Finland | **Clinical Groups** | **Odontology Group** |
| Aino Salminen | Hospital District of Helsinki and Uusimaa, Helsinki, Finland | **Clinical Groups** | **Odontology Group** |
| Tuula Salo | Hospital District of Helsinki and Uusimaa, Helsinki, Finland | **Clinical Groups** | **Odontology Group** |
| David Rice | Hospital District of Helsinki and Uusimaa, Helsinki, Finland | **Clinical Groups** | **Odontology Group** |
| Pekka Nieminen | Hospital District of Helsinki and Uusimaa, Helsinki, Finland | **Clinical Groups** | **Odontology Group** |
| Ulla Palotie | Hospital District of Helsinki and Uusimaa, Helsinki, Finland | **Clinical Groups** | **Odontology Group** |
| Maria Siponen | Northern Savo Hospital District, Kuopio, Finland | **Clinical Groups** | **Odontology Group** |
| Liisa Suominen | Northern Savo Hospital District, Kuopio, Finland | **Clinical Groups** | **Odontology Group** |
| Päivi Mäntylä | Northern Savo Hospital District, Kuopio, Finland | **Clinical Groups** | **Odontology Group** |
| Ulvi Gursoy | Hospital District of Southwest Finland, Turku, Finland | **Clinical Groups** | **Odontology Group** |
| Vuokko Anttonen | Northern Ostrobothnia Hospital District, Oulu, Finland | **Clinical Groups** | **Odontology Group** |
| Kirsi Sipilä | Research Unit of Oral Health Sciences Faculty of Medicine, University of Oulu, Oulu, Finland; Medical Research Center, Oulu, Oulu University Hospital and University of Oulu, Oulu, Finland | **Clinical Groups** | **Odontology Group** |
| Rion Pendergrass | Genentech, San Francisco, CA, United States | **Clinical Groups** | **Odontology Group** |
| Hannele Laivuori | Institute for Molecular Medicine Finland (FIMM), HiLIFE, University of Helsinki, Helsinki, Finland | **Clinical Groups** | **Women’s Health and Reproduction Group** |
| Venla Kurra | Pirkanmaa Hospital District, Tampere, Finland | **Clinical Groups** | **Women’s Health and Reproduction Group** |
| Laura Kotaniemi-Talonen | Pirkanmaa Hospital District, Tampere, Finland | **Clinical Groups** | **Women’s Health and Reproduction Group** |
| Oskari Heikinheimo | Hospital District of Helsinki and Uusimaa, Helsinki, Finland | **Clinical Groups** | **Women’s Health and Reproduction Group** |
| Ilkka Kalliala | Hospital District of Helsinki and Uusimaa, Helsinki, Finland | **Clinical Groups** | **Women’s Health and Reproduction Group** |
| Lauri Aaltonen | Hospital District of Helsinki and Uusimaa, Helsinki, Finland | **Clinical Groups** | **Women’s Health and Reproduction Group** |
| Varpu Jokimaa | Hospital District of Southwest Finland, Turku, Finland | **Clinical Groups** | **Women’s Health and Reproduction Group** |
| Johannes Kettunen | Northern Ostrobothnia Hospital District, Oulu, Finland | **Clinical Groups** | **Women’s Health and Reproduction Group** |
| Marja Vääräsmäki | Northern Ostrobothnia Hospital District, Oulu, Finland | **Clinical Groups** | **Women’s Health and Reproduction Group** |
| Outi Uimari | Northern Ostrobothnia Hospital District, Oulu, Finland | **Clinical Groups** | **Women’s Health and Reproduction Group** |
| Laure Morin-Papunen | Northern Ostrobothnia Hospital District, Oulu, Finland | **Clinical Groups** | **Women’s Health and Reproduction Group** |
| Maarit Niinimäki | Northern Ostrobothnia Hospital District, Oulu, Finland | **Clinical Groups** | **Women’s Health and Reproduction Group** |
| Terhi Piltonen | Northern Ostrobothnia Hospital District, Oulu, Finland | **Clinical Groups** | **Women’s Health and Reproduction Group** |
| Katja Kivinen | Institute for Molecular Medicine Finland (FIMM), HiLIFE, University of Helsinki, Helsinki, Finland | **Clinical Groups** | **Women’s Health and Reproduction Group** |
| Elisabeth Widen | Institute for Molecular Medicine Finland (FIMM), HiLIFE, University of Helsinki, Helsinki, Finland | **Clinical Groups** | **Women’s Health and Reproduction Group** |
| Taru Tukiainen | Institute for Molecular Medicine Finland (FIMM), HiLIFE, University of Helsinki, Helsinki, Finland | **Clinical Groups** | **Women’s Health and Reproduction Group** |
| Mary Pat Reeve | Institute for Molecular Medicine Finland (FIMM), HiLIFE, University of Helsinki, Helsinki, Finland | **Clinical Groups** | **Women’s Health and Reproduction Group** |
| Mark Daly | Institute for Molecular Medicine Finland (FIMM), HiLIFE, University of Helsinki, Helsinki, Finland; Broad Institute of MIT and Harvard; Massachusetts General Hospital | **Clinical Groups** | **Women’s Health and Reproduction Group** |
| Niko Välimäki | University of Helsinki, Helsinki, Finland | **Clinical Groups** | **Women’s Health and Reproduction Group** |
| Eija Laakkonen | University of Jyväskylä, Jyväskylä, Finland | **Clinical Groups** | **Women’s Health and Reproduction Group** |
| Jaakko Tyrmi | University of Oulu, Oulu, Finland / University of Tampere, Tampere, Finland | **Clinical Groups** | **Women’s Health and Reproduction Group** |
| Heidi Silven | University of Oulu, Oulu, Finland | **Clinical Groups** | **Women’s Health and Reproduction Group** |
| Eeva Sliz | University of Oulu, Oulu, Finland | **Clinical Groups** | **Women’s Health and Reproduction Group** |
| Riikka Arffman | University of Oulu, Oulu, Finland | **Clinical Groups** | **Women’s Health and Reproduction Group** |
| Susanna Savukoski | University of Oulu, Oulu, Finland | **Clinical Groups** | **Women’s Health and Reproduction Group** |
| Triin Laisk | Estonian biobank, Tartu, Estonia | **Clinical Groups** | **Women’s Health and Reproduction Group** |
| Natalia Pujol | Estonian biobank, Tartu, Estonia | **Clinical Groups** | **Women’s Health and Reproduction Group** |
| Mengzhen Liu | Abbvie, Chicago, IL, United States | **Clinical Groups** | **Women’s Health and Reproduction Group** |
| Bridget Riley-Gillis | Abbvie, Chicago, IL, United States | **Clinical Groups** | **Women’s Health and Reproduction Group** |
| Rion Pendergrass | Genentech, San Francisco, CA, United States | **Clinical Groups** | **Women’s Health and Reproduction Group** |
| Janet Kumar | GlaxoSmithKline, Collegeville, PA, United States | **Clinical Groups** | **Women’s Health and Reproduction Group** |
| Kirsi Auro | GlaxoSmithKline, Espoo, Finland | **Clinical Groups** | **Women’s Health and Reproduction Group** |
| Iiris Hovatta | University of Helsinki, Finland | **Clinical Groups** | **Depression group** |
| Chia-Yen Chen | Biogen, Cambridge, MA, United States | **Clinical Groups** | **Depression group** |
| Erkki Isometsä | Hospital District of Helsinki and Uusimaa, Helsinki, Finland | **Clinical Groups** | **Depression group** |
| Hanna Ollila | Institute for Molecular Medicine Finland (FIMM), HiLIFE, University of Helsinki, Helsinki, Finland | **Clinical Groups** | **Depression group** |
| Jaana Suvisaari | Finnish Institute for Health and Welfare (THL), Helsinki, Finland | **Clinical Groups** | **Depression group** |
| Thomas Damm Als | Aarhus University, Denmark | **Clinical Groups** | **Depression group** |
| Antti Mäkitie | Department of Otorhinolaryngology - Head and Neck Surgery, University of Helsinki and Helsinki University Hospital, Helsinki, Finland | **Clinical Groups** | **ENT (ear, nose and throath) Group** |
| Argyro Bizaki-Vallaskangas | Pirkanmaa Hospital District, Tampere, Finland | **Clinical Groups** | **ENT (ear, nose and throath) Group** |
| Sanna Toppila-Salmi | University of Eastern Finland and Kuopio University Hospital, Department of Otorhinolaryngology, Kuopio, Finland and Department of Allergy, Helsinki University Hospital and University of Helsinki, Finland | **Clinical Groups** | **ENT (ear, nose and throath) Group** |
| Tytti Willberg | Hospital District of Southwest Finland, Turku, Finland | **Clinical Groups** | **ENT (ear, nose and throath) Group** |
| Elmo Saarentaus | Institute for Molecular Medicine Finland (FIMM), HiLIFE, University of Helsinki, Helsinki, Finland | **Clinical Groups** | **ENT (ear, nose and throath) Group** |
| Antti Aarnisalo | Hospital District of Helsinki and Uusimaa, Helsinki, Finland | **Clinical Groups** | **ENT (ear, nose and throath) Group** |
| Eveliina Salminen | Hospital District of Helsinki and Uusimaa, Helsinki, Finland | **Clinical Groups** | **ENT (ear, nose and throath) Group** |
| Elisa Rahikkala | Northern Ostrobothnia Hospital District, Oulu, Finland | **Clinical Groups** | **ENT (ear, nose and throath) Group** |
| Johannes Kettunen | Northern Ostrobothnia Hospital District, Oulu, Finland | **Clinical Groups** | **ENT (ear, nose and throath) Group** |
| Kristiina Aittomäki | Department of Medical Genetics, Helsinki University Central Hospital, Helsinki, Finland | **Clinical Groups** | **POI (premature ovarian failure) Group** |
| Fredrik Åberg | Transplantation and Liver Surgery Clinic, Helsinki University Hospital, Helsinki University, Helsinki, Finland | **Clinical Groups** | **LiverScore Group** |
| Mitja Kurki | Institute for Molecular Medicine Finland (FIMM), HiLIFE, University of Helsinki, Helsinki, Finland; Broad Institute, Cambridge, MA, United States | **FinnGen Analysis working group** | **FinnGen Analysis working group** |
| Samuli Ripatti | Institute for Molecular Medicine Finland (FIMM), HiLIFE, University of Helsinki, Helsinki, Finland | **FinnGen Analysis working group** | **FinnGen Analysis working group** |
| Mark Daly | Institute for Molecular Medicine, Finland (FIMM), HiLIFE, University of Helsinki, Helsinki, Finland; Broad Institute of MIT and Harvard; Massachusetts General Hospital | **FinnGen Analysis working group** | **FinnGen Analysis working group** |
| Juha Karjalainen | Institute for Molecular Medicine Finland (FIMM), HiLIFE, University of Helsinki, Helsinki, Finland | **FinnGen Analysis working group** | **FinnGen Analysis working group** |
| Aki Havulinna | Institute for Molecular Medicine Finland (FIMM), HiLIFE, University of Helsinki, Helsinki, Finland; Finnish Institute for Health and Welfare (THL), Helsinki, Finland | **FinnGen Analysis working group** | **FinnGen Analysis working group** |
| Juha Mehtonen | Institute for Molecular Medicine Finland (FIMM), HiLIFE, University of Helsinki, Helsinki, Finland | **FinnGen Analysis working group** | **FinnGen Analysis working group** |
| Priit Palta | Institute for Molecular Medicine Finland (FIMM), HiLIFE, University of Helsinki, Helsinki, Finland | **FinnGen Analysis working group** | **FinnGen Analysis working group** |
| Shabbeer Hassan | Institute for Molecular Medicine Finland (FIMM), HiLIFE, University of Helsinki, Helsinki, Finland | **FinnGen Analysis working group** | **FinnGen Analysis working group** |
| Pietro Della Briotta Parolo | Institute for Molecular Medicine Finland (FIMM), HiLIFE, University of Helsinki, Helsinki, Finland | **FinnGen Analysis working group** | **FinnGen Analysis working group** |
| Wei Zhou | Broad Institute, Cambridge, MA, United States | **FinnGen Analysis working group** | **FinnGen Analysis working group** |
| Mutaamba Maasha | Broad Institute, Cambridge, MA, United States | **FinnGen Analysis working group** | **FinnGen Analysis working group** |
| Shabbeer Hassan | Institute for Molecular Medicine Finland (FIMM), HiLIFE, University of Helsinki, Helsinki, Finland | **FinnGen Analysis working group** | **FinnGen Analysis working group** |
| Susanna Lemmelä | Institute for Molecular Medicine Finland (FIMM), HiLIFE, University of Helsinki, Helsinki, Finland | **FinnGen Analysis working group** | **FinnGen Analysis working group** |
| Manuel Rivas | University of Stanford, Stanford, CA, United States | **FinnGen Analysis working group** | **FinnGen Analysis working group** |
| Aarno Palotie | Institute for Molecular Medicine Finland (FIMM), HiLIFE, University of Helsinki, Helsinki, Finland | **FinnGen Analysis working group** | **FinnGen Analysis working group** |
| Aoxing Liu | Institute for Molecular Medicine Finland (FIMM), HiLIFE, University of Helsinki, Helsinki, Finland | **FinnGen Analysis working group** | **FinnGen Analysis working group** |
| Arto Lehisto | Institute for Molecular Medicine Finland (FIMM), HiLIFE, University of Helsinki, Helsinki, Finland | **FinnGen Analysis working group** | **FinnGen Analysis working group** |
| Andrea Ganna | Institute for Molecular Medicine Finland (FIMM), HiLIFE, University of Helsinki, Helsinki, Finland | **FinnGen Analysis working group** | **FinnGen Analysis working group** |
| Vincent Llorens | Institute for Molecular Medicine Finland (FIMM), HiLIFE, University of Helsinki, Helsinki, Finland | **FinnGen Analysis working group** | **FinnGen Analysis working group** |
| Hannele Laivuori | Institute for Molecular Medicine Finland (FIMM), HiLIFE, University of Helsinki, Helsinki, Finland | **FinnGen Analysis working group** | **FinnGen Analysis working group** |
| Taru Tukiainen | Institute for Molecular Medicine Finland (FIMM), HiLIFE, University of Helsinki, Helsinki, Finland | **FinnGen Analysis working group** | **FinnGen Analysis working group** |
| Mary Pat Reeve | Institute for Molecular Medicine Finland (FIMM), HiLIFE, University of Helsinki, Helsinki, Finland | **FinnGen Analysis working group** | **FinnGen Analysis working group** |
| Henrike Heyne | Institute for Molecular Medicine Finland (FIMM), HiLIFE, University of Helsinki, Helsinki, Finland | **FinnGen Analysis working group** | **FinnGen Analysis working group** |
| Nina Mars | Institute for Molecular Medicine Finland (FIMM), HiLIFE, University of Helsinki, Helsinki, Finland | **FinnGen Analysis working group** | **FinnGen Analysis working group** |
| Joel Rämö | Institute for Molecular Medicine Finland (FIMM), HiLIFE, University of Helsinki, Helsinki, Finland | **FinnGen Analysis working group** | **FinnGen Analysis working group** |
| Elmo Saarentaus | Institute for Molecular Medicine Finland (FIMM), HiLIFE, University of Helsinki, Helsinki, Finland | **FinnGen Analysis working group** | **FinnGen Analysis working group** |
| Hanna Ollila | Institute for Molecular Medicine Finland (FIMM), HiLIFE, University of Helsinki, Helsinki, Finland | **FinnGen Analysis working group** | **FinnGen Analysis working group** |
| Rodos Rodosthenous | Institute for Molecular Medicine Finland (FIMM), HiLIFE, University of Helsinki, Helsinki, Finland | **FinnGen Analysis working group** | **FinnGen Analysis working group** |
| Satu Strausz | Institute for Molecular Medicine Finland (FIMM), HiLIFE, University of Helsinki, Helsinki, Finland | **FinnGen Analysis working group** | **FinnGen Analysis working group** |
| Tuula Palotie | University of Helsinki and Hospital District of Helsinki and Uusimaa, Helsinki, Finland | **FinnGen Analysis working group** | **FinnGen Analysis working group** |
| Kimmo Palin | University of Helsinki, Helsinki, Finland | **FinnGen Analysis working group** | **FinnGen Analysis working group** |
| Javier Garcia-Tabuenca | University of Tampere, Tampere, Finland | **FinnGen Analysis working group** | **FinnGen Analysis working group** |
| Harri Siirtola | University of Tampere, Tampere, Finland | **FinnGen Analysis working group** | **FinnGen Analysis working group** |
| Tuomo Kiiskinen | Institute for Molecular Medicine Finland (FIMM), HiLIFE, University of Helsinki, Helsinki, Finland | **FinnGen Analysis working group** | **FinnGen Analysis working group** |
| Jiwoo Lee | Institute for Molecular Medicine Finland (FIMM), HiLIFE, University of Helsinki, Helsinki, Finland; Broad Institute, Cambridge, MA, United States | **FinnGen Analysis working group** | **FinnGen Analysis working group** |
| Kristin Tsuo | Institute for Molecular Medicine Finland (FIMM), HiLIFE, University of Helsinki, Helsinki, Finland; Broad Institute, Cambridge, MA, United States | **FinnGen Analysis working group** | **FinnGen Analysis working group** |
| Amanda Elliott | Institute for Molecular Medicine Finland (FIMM), HiLIFE, University of Helsinki, Helsinki, Finland; Broad Institute, Cambridge, MA, USA and Massachusetts General Hospital, Boston, MA, USA | **FinnGen Analysis working group** | **FinnGen Analysis working group** |
| Kati Kristiansson | THL Biobank / Finnish Institute for Health and Welfare (THL), Helsinki, Finland | **FinnGen Analysis working group** | **FinnGen Analysis working group** |
| Mikko Arvas | Finnish Red Cross Blood Service / Finnish Hematology Registry and Clinical Biobank, Helsinki, Finland | **FinnGen Analysis working group** | **FinnGen Analysis working group** |
| Kati Hyvärinen | Finnish Red Cross Blood Service, Helsinki, Finland | **FinnGen Analysis working group** | **FinnGen Analysis working group** |
| Jarmo Ritari | Finnish Red Cross Blood Service, Helsinki, Finland | **FinnGen Analysis working group** | **FinnGen Analysis working group** |
| Olli Carpén | Helsinki Biobank / Helsinki University and Hospital District of Helsinki and Uusimaa, Helsinki | **FinnGen Analysis working group** | **FinnGen Analysis working group** |
| Johannes Kettunen | Northern Finland Biobank Borealis / University of Oulu / Northern Ostrobothnia Hospital District, Oulu, Finland | **FinnGen Analysis working group** | **FinnGen Analysis working group** |
| Katri Pylkäs | University of Oulu, Oulu, Finland | **FinnGen Analysis working group** | **FinnGen Analysis working group** |
| Eeva Sliz | University of Oulu, Oulu, Finland | **FinnGen Analysis working group** | **FinnGen Analysis working group** |
| Minna Karjalainen | University of Oulu, Oulu, Finland | **FinnGen Analysis working group** | **FinnGen Analysis working group** |
| Tuomo Mantere | Northern Finland Biobank Borealis / University of Oulu / Northern Ostrobothnia Hospital District, Oulu, Finland | **FinnGen Analysis working group** | **FinnGen Analysis working group** |
| Eeva Kangasniemi | Finnish Clinical Biobank Tampere / University of Tampere / Pirkanmaa Hospital District, Tampere, Finland | **FinnGen Analysis working group** | **FinnGen Analysis working group** |
| Sami Heikkinen | University of Eastern Finland, Kuopio, Finland | **FinnGen Analysis working group** | **FinnGen Analysis working group** |
| Arto Mannermaa | Biobank of Eastern Finland / University of Eastern Finland / Northern Savo Hospital District, Kuopio, Finland | **FinnGen Analysis working group** | **FinnGen Analysis working group** |
| Eija Laakkonen | University of Jyväskylä, Jyväskylä, Finland | **FinnGen Analysis working group** | **FinnGen Analysis working group** |
| Nina Pitkänen | Auria Biobank / University of Turku / Hospital District of Southwest Finland, Turku, Finland | **FinnGen Analysis working group** | **FinnGen Analysis working group** |
| Samuel Lessard | Translational Sciences, Sanofi R&D, Framingham, MA, USA | **FinnGen Analysis working group** | **FinnGen Analysis working group** |
| Clément Chatelain | Translational Sciences, Sanofi R&D, Framingham, MA, USA | **FinnGen Analysis working group** | **FinnGen Analysis working group** |
| Lila Kallio | Auria Biobank / University of Turku / Hospital District of Southwest Finland, Turku, Finland | **Biobank directors** | **Biobank directors** |
| Tiina Wahlfors | THL Biobank / Finnish Institute for Health and Welfare (THL), Helsinki, Finland | **Biobank directors** | **Biobank directors** |
| Jukka Partanen | Finnish Red Cross Blood Service / Finnish Hematology Registry and Clinical Biobank, Helsinki, Finland | **Biobank directors** | **Biobank directors** |
| Eero Punkka | Helsinki Biobank / Helsinki University and Hospital District of Helsinki and Uusimaa, Helsinki | **Biobank directors** | **Biobank directors** |
| Raisa Serpi | Northern Finland Biobank Borealis / University of Oulu / Northern Ostrobothnia Hospital District, Oulu, Finland | **Biobank directors** | **Biobank directors** |
| Sanna Siltanen | Finnish Clinical Biobank Tampere / University of Tampere / Pirkanmaa Hospital District, Tampere, Finland | **Biobank directors** | **Biobank directors** |
| Veli-Matti Kosma | Biobank of Eastern Finland / University of Eastern Finland / Northern Savo Hospital District, Kuopio, Finland | **Biobank directors** | **Biobank directors** |
| Teijo Kuopio | Central Finland Biobank / University of Jyväskylä / Central Finland Health Care District, Jyväskylä, Finland | **Biobank directors** | **Biobank directors** |
| Anu Jalanko | Institute for Molecular Medicine Finland (FIMM), HiLIFE, University of Helsinki, Helsinki, Finland | **FinnGen Teams** | **Administration** |
| Huei-Yi Shen | Institute for Molecular Medicine Finland (FIMM), HiLIFE, University of Helsinki, Helsinki, Finland | **FinnGen Teams** | **Administration** |
| Risto Kajanne | Institute for Molecular Medicine Finland (FIMM), HiLIFE, University of Helsinki, Helsinki, Finland | **FinnGen Teams** | **Administration** |
| Mervi Aavikko | Institute for Molecular Medicine Finland (FIMM), HiLIFE, University of Helsinki, Helsinki, Finland | **FinnGen Teams** | **Administration** |
| Helen Cooper | Institute for Molecular Medicine Finland (FIMM), HiLIFE, University of Helsinki, Helsinki, Finland | **FinnGen Teams** | **Administration** |
| Denise Öller | Institute for Molecular Medicine Finland (FIMM), HiLIFE, University of Helsinki, Helsinki, Finland | **FinnGen Teams** | **Administration** |
| Rasko Leinonen | Institute for Molecular Medicine Finland (FIMM), HiLIFE, University of Helsinki, Helsinki, Finland; European Molecular Biology Laboratory, European Bioinformatics Institute, Cambridge, UK | **FinnGen Teams** | **Administration** |
| Henna Palin | Finnish Clinical Biobank Tampere / University of Tampere / Pirkanmaa Hospital District, Tampere, Finland | **FinnGen Teams** | **Administration** |
| Malla-Maria Linna | Helsinki Biobank / Helsinki University and Hospital District of Helsinki and Uusimaa, Helsinki | **FinnGen Teams** | **Administration** |
| Mitja Kurki | Institute for Molecular Medicine Finland (FIMM), HiLIFE, University of Helsinki, Helsinki, Finland; Broad Institute, Cambridge, MA, United States | **FinnGen Teams** | **Analysis** |
| Juha Karjalainen | Institute for Molecular Medicine Finland (FIMM), HiLIFE, University of Helsinki, Helsinki, Finland | **FinnGen Teams** | **Analysis** |
| Pietro Della Briotta Parolo | Institute for Molecular Medicine Finland (FIMM), HiLIFE, University of Helsinki, Helsinki, Finland | **FinnGen Teams** | **Analysis** |
| Arto Lehisto | Institute for Molecular Medicine Finland (FIMM), HiLIFE, University of Helsinki, Helsinki, Finland | **FinnGen Teams** | **Analysis** |
| Juha Mehtonen | Institute for Molecular Medicine Finland (FIMM), HiLIFE, University of Helsinki, Helsinki, Finland | **FinnGen Teams** | **Analysis** |
| Wei Zhou | Broad Institute, Cambridge, MA, United States | **FinnGen Teams** | **Analysis** |
| Masahiro Kanai | Broad Institute, Cambridge, MA, United States | **FinnGen Teams** | **Analysis** |
| Mutaamba Maasha | Broad Institute, Cambridge, MA, United States | **FinnGen Teams** | **Analysis** |
| Zhili Zheng | Broad Institute, Cambridge, MA, United States | **FinnGen Teams** | **Analysis** |
| Hannele Laivuori | Institute for Molecular Medicine Finland (FIMM), HiLIFE, University of Helsinki, Helsinki, Finland | **FinnGen Teams** | **Clinical Endpoint Development** |
| Aki Havulinna | Institute for Molecular Medicine Finland (FIMM), HiLIFE, University of Helsinki, Helsinki, Finland; Finnish Institute for Health and Welfare (THL), Helsinki, Finland | **FinnGen Teams** | **Clinical Endpoint Development** |
| Susanna Lemmelä | Institute for Molecular Medicine Finland (FIMM), HiLIFE, University of Helsinki, Helsinki, Finland | **FinnGen Teams** | **Clinical Endpoint Development** |
| Tuomo Kiiskinen | Institute for Molecular Medicine Finland (FIMM), HiLIFE, University of Helsinki, Helsinki, Finland | **FinnGen Teams** | **Clinical Endpoint Development** |
| L. Elisa Lahtela | Institute for Molecular Medicine Finland (FIMM), HiLIFE, University of Helsinki, Helsinki, Finland | **FinnGen Teams** | **Clinical Endpoint Development** |
| Mari Kaunisto | Institute for Molecular Medicine Finland (FIMM), HiLIFE, University of Helsinki, Helsinki, Finland | **FinnGen Teams** | **Communication** |
| Elina Kilpeläinen | Institute for Molecular Medicine Finland (FIMM), HiLIFE, University of Helsinki, Helsinki, Finland | **FinnGen Teams** | **E-Science** |
| Timo P. Sipilä | Institute for Molecular Medicine Finland (FIMM), HiLIFE, University of Helsinki, Helsinki, Finland | **FinnGen Teams** | **E-Science** |
| Oluwaseun Alexander Dada | Institute for Molecular Medicine Finland (FIMM), HiLIFE, University of Helsinki, Helsinki, Finland | **FinnGen Teams** | **E-Science** |
| Awaisa Ghazal | Institute for Molecular Medicine Finland (FIMM), HiLIFE, University of Helsinki, Helsinki, Finland | **FinnGen Teams** | **E-Science** |
| Anastasia Kytölä | Institute for Molecular Medicine Finland (FIMM), HiLIFE, University of Helsinki, Helsinki, Finland | **FinnGen Teams** | **E-Science** |
| Rigbe Weldatsadik | Institute for Molecular Medicine Finland (FIMM), HiLIFE, University of Helsinki, Helsinki, Finland | **FinnGen Teams** | **E-Science** |
| Sanni Ruotsalainen | Institute for Molecular Medicine Finland (FIMM), HiLIFE, University of Helsinki, Helsinki, Finland | **FinnGen Teams** | **E-Science** |
| Kati Donner | Institute for Molecular Medicine Finland (FIMM), HiLIFE, University of Helsinki, Helsinki, Finland | **FinnGen Teams** | **Genotyping** |
| Timo P. Sipilä | Institute for Molecular Medicine Finland (FIMM), HiLIFE, University of Helsinki, Helsinki, Finland | **FinnGen Teams** | **Genotyping** |
| Anu Loukola | Helsinki Biobank / Helsinki University and Hospital District of Helsinki and Uusimaa, Helsinki | **FinnGen Teams** | **Sample Collection Coordination** |
| Päivi Laiho | THL Biobank / Finnish Institute for Health and Welfare (THL), Helsinki, Finland | **FinnGen Teams** | **Sample Logistics** |
| Tuuli Sistonen | THL Biobank / Finnish Institute for Health and Welfare (THL), Helsinki, Finland | **FinnGen Teams** | **Sample Logistics** |
| Essi Kaiharju | THL Biobank / Finnish Institute for Health and Welfare (THL), Helsinki, Finland | **FinnGen Teams** | **Sample Logistics** |
| Markku Laukkanen | THL Biobank / Finnish Institute for Health and Welfare (THL), Helsinki, Finland | **FinnGen Teams** | **Sample Logistics** |
| Elina Järvensivu | THL Biobank / Finnish Institute for Health and Welfare (THL), Helsinki, Finland | **FinnGen Teams** | **Sample Logistics** |
| Sini Lähteenmäki | THL Biobank / Finnish Institute for Health and Welfare (THL), Helsinki, Finland | **FinnGen Teams** | **Sample Logistics** |
| Lotta Männikkö | THL Biobank / Finnish Institute for Health and Welfare (THL), Helsinki, Finland | **FinnGen Teams** | **Sample Logistics** |
| Regis Wong | THL Biobank / Finnish Institute for Health and Welfare (THL), Helsinki, Finland | **FinnGen Teams** | **Sample Logistics** |
| Auli Toivola | THL Biobank / Finnish Institute for Health and Welfare (THL), Helsinki, Finland | **FinnGen Teams** | **Sample Logistics** |
| Minna Brunfeldt | THL Biobank / Finnish Institute for Health and Welfare (THL), Helsinki, Finland | **FinnGen Teams** | **Registry Data Operations** |
| Hannele Mattsson | THL Biobank / Finnish Institute for Health and Welfare (THL), Helsinki, Finland | **FinnGen Teams** | **Registry Data Operations** |
| Kati Kristiansson | THL Biobank / Finnish Institute for Health and Welfare (THL), Helsinki, Finland | **FinnGen Teams** | **Registry Data Operations** |
| Susanna Lemmelä | Institute for Molecular Medicine Finland (FIMM), HiLIFE, University of Helsinki, Helsinki, Finland | **FinnGen Teams** | **Registry Data Operations** |
| Sami Koskelainen | THL Biobank / Finnish Institute for Health and Welfare (THL), Helsinki, Finland | **FinnGen Teams** | **Registry Data Operations** |
| Tero Hiekkalinna | THL Biobank / Finnish Institute for Health and Welfare (THL), Helsinki, Finland | **FinnGen Teams** | **Registry Data Operations** |
| Teemu Paajanen | THL Biobank / Finnish Institute for Health and Welfare (THL), Helsinki, Finland | **FinnGen Teams** | **Registry Data Operations** |
| Priit Palta | Institute for Molecular Medicine Finland (FIMM), HiLIFE, University of Helsinki, Helsinki, Finland | **FinnGen Teams** | **Sequencing Informatics** |
| Kalle Pärn | Institute for Molecular Medicine Finland (FIMM), HiLIFE, University of Helsinki, Helsinki, Finland | **FinnGen Teams** | **Sequencing Informatics** |
| Mart Kals | Institute for Molecular Medicine Finland (FIMM), HiLIFE, University of Helsinki, Helsinki, Finland | **FinnGen Teams** | **Sequencing Informatics** |
| Shuang Luo | Institute for Molecular Medicine Finland (FIMM), HiLIFE, University of Helsinki, Helsinki, Finland | **FinnGen Teams** | **Sequencing Informatics** |
| Tarja Laitinen | Pirkanmaa Hospital District, Tampere, Finland | **FinnGen Teams** | **Trajectory** |
| Mary Pat Reeve | Institute for Molecular Medicine Finland (FIMM), HiLIFE, University of Helsinki, Helsinki, Finland | **FinnGen Teams** | **Trajectory** |
| Shanmukha Sampath Padmanabhuni | Institute for Molecular Medicine Finland (FIMM), HiLIFE, University of Helsinki, Helsinki, Finland | **FinnGen Teams** | **Trajectory** |
| Marianna Niemi | University of Tampere, Tampere, Finland | **FinnGen Teams** | **Trajectory** |
| Harri Siirtola | University of Tampere, Tampere, Finland | **FinnGen Teams** | **Trajectory** |
| Javier Gracia-Tabuenca | University of Tampere, Tampere, Finland | **FinnGen Teams** | **Trajectory** |
| Mika Helminen | University of Tampere, Tampere, Finland | **FinnGen Teams** | **Trajectory** |
| Tiina Luukkaala | University of Tampere, Tampere, Finland | **FinnGen Teams** | **Trajectory** |
| Iida Vähätalo | University of Tampere, Tampere, Finland | **FinnGen Teams** | **Trajectory** |
| Jyrki Tammerluoto | Institute for Molecular Medicine Finland (FIMM), HiLIFE, University of Helsinki, Helsinki, Finland | **FinnGen Teams** | **Data protection officer** |
| Marco Hautalahti | Finnish Biobank Cooperative - FINBB | **FinnGen Teams** | **FINBB - Finnish biobank cooperative** |
| Johanna Mäkelä | Finnish Biobank Cooperative - FINBB | **FinnGen Teams** | **FINBB - Finnish biobank cooperative** |
| Sarah Smith | Finnish Biobank Cooperative - FINBB | **FinnGen Teams** | **FINBB - Finnish biobank cooperative** |
| Tom Southerington | Finnish Biobank Cooperative - FINBB | **FinnGen Teams** | **FINBB - Finnish biobank cooperative** |
| Petri Lehto | Finnish Biobank Cooperative - FINBB | **FinnGen Teams** | **FINBB - Finnish biobank cooperative** |

**Table S2.** A list of Estonian Biobank Research Team members and their affiliations

| Full Name | Affiliation |
| --- | --- |
| Andres Metspalu | Estonian Genome Centre, Institute of Genomics, University of Tartu, Tartu, Estonia |
| Mari Nelis | Estonian Genome Centre, Institute of Genomics, University of Tartu, Tartu, Estonia |
| Lili Milani | Estonian Genome Centre, Institute of Genomics, University of Tartu, Tartu, Estonia |
| Reedik Mägi | Estonian Genome Centre, Institute of Genomics, University of Tartu, Tartu, Estonia |
| Georgi Hudjashov | Estonian Genome Centre, Institute of Genomics, University of Tartu, Tartu, Estonia |
| Tõnu Esko | Estonian Genome Centre, Institute of Genomics, University of Tartu, Tartu, Estonia |

## **Table S3.** The lead variants of the SNHL-associated loci (P<5x10^-8^, we defined the locus with a 1 MB window) identified in the genome-wide meta-analysis focusing on general population of 35 960 SNHL cases and 495 099 controls from the FinnGen and the Estonian Biobank.  Loci with a grey background were located in the HLA region and were counted as one locus.

| **RSID** | **CHR:POS (hg38)** | **EA/NEA** | **Population** | **EAF** | **OR (95% CI)** | **P** | **HetPVal** | **Fin Enric.** |
| --- | --- | --- | --- | --- | --- | --- | --- | --- |
| rs72660919 | 1:25351114 | A/G | Meta | 0.134 | 1.070 (1.046-1.095)  1.048 (0.998-1.100)  1.077 (1.049-1.106) | 1.10x10^-8^  0.058  4.33x10^-8^ | 0.337 | 1.015 |
|  |  |  | EstBB | 0.128 |  |  |  |  |
|  |  |  | FinnGen | 0.137 |  |  |  |  |
|  |  |  | European | 0.135 |  |  |  |  |
| rs6690926 | 1:45669306 | T/C | Meta | 0.721 | 0.952 (0.935-0.969)  0.930 (0.898-0.963)  0.959 (0.940-0.979) | 3.94x10^-8^  4.87x10^-5^  6.66x10^-5^ | 0.136 | 1.031 |
|  |  |  | EstBB | 0.711 |  |  |  |  |
|  |  |  | FinnGen | 0.725 |  |  |  |  |
|  |  |  | European | 0.703 |  |  |  |  |
| rs10176445 | 2:54618639 | C/G | Meta | 0.186 | 1.098 (1.076-1.121)  1.052 (1.011-1.094)  1.115 (1.089-1.141) | 9.88x10^-20^  0.013  9.80x10^-20^ | 0.013 | 0.784 |
|  |  |  | EstBB | 0.202 |  |  |  |  |
|  |  |  | FinnGen | 0.178 |  |  |  |  |
|  |  |  | European | 0.227 |  |  |  |  |
| rs4680280 | 3:156532919 | A/G | Meta | 0.826 | 1.065 (1.042-1.087)  1.057 (1.014-1.102)  1.067 (1.041-1.094) | 7.24x10^-9^  0.009  2.37x10^-7^ | 0.712 | 1.021 |
|  |  |  | EstBB | 0.816 |  |  |  |  |
|  |  |  | FinnGen | 0.832 |  |  |  |  |
|  |  |  | European | 0.815 |  |  |  |  |
| rs72622586 | 3:182277016 | A/G | Meta | 0.802 | 1.066 (1.045-1.088)  1.046 (1.006-1.088)  1.074 (1.049-1.100) | 5.67x10^-10^  0.024  4.08x10^-9^ | 0.258 | 1.014 |
|  |  |  | EstBB | 0.776 |  |  |  |  |
|  |  |  | FinnGen | 0.815 |  |  |  |  |
|  |  |  | European | 0.804 |  |  |  |  |
| rs4704097 | 5:73773756 | A/G | Meta | 0.465 | 0.941 (0.926-0.956)  0.960 (0.929-0.992)  0.935 (0.918-0.953) | 1.50x10^-13^  0.013  1.34x10^-12^ | 0.180 | 1.009 |
|  |  |  | EstBB | 0.481 |  |  |  |  |
|  |  |  | FinnGen | 0.457 |  |  |  |  |
|  |  |  | European | 0.453 |  |  |  |  |
| rs201654808 | 6:26430520 | CA/C | Meta | 0.980 | 0.852 (0.808-0.900)  0.957 (0.811-1.129)  0.841 (0.795-0.890) | 5.46x10^-9^  0.604  2.12x10^-9^ | 0.146 | 0.980 |
|  |  |  | EstBB | 0.990 |  |  |  |  |
|  |  |  | FinnGen | 0.974 |  |  |  |  |
|  |  |  | European | 0.994 |  |  |  |  |
| rs146891690 | 6:27549594 | A/G | Meta | 0.030 | 1.178 (1.126-1.232)  1.122 (1.012-1.245)  1.191 (1.133-1.252) | 8.66x10^-13^  0.029  5.80x10-^12^ | 0.312 | 4.857 |
|  |  |  | EstBB | 0.023 |  |  |  |  |
|  |  |  | FinnGen | 0.034 |  |  |  |  |
|  |  |  | European | 0.007 |  |  |  |  |
| rs187298284 | 6:29570199 | T/C | Meta | 0.017 | 1.207 (1.138-1.279)  1.038 (0.818-1.317)  1.218 (1.147-1.294) | 2.83x1^0-10^  0.757  1.29x10^-10^ | 0.201 | 46.00 |
|  |  |  | EstBB | 0.005 |  |  |  |  |
|  |  |  | FinnGen | 0.023 |  |  |  |  |
|  |  |  | European | 0.0005 |  |  |  |  |
| rs193150789 | 6:30914729 | T/C | Meta | 0.981 | 0.812 (0.769-0.858)  0.967 (0.814-1.147)  0.796 (0.751-0.843) | 9.90x10^-14^  0.697  1.13x10^-14^ | 0.035 | 0.978 |
|  |  |  | EstBB | 0.991 |  |  |  |  |
|  |  |  | FinnGen | 0.976 |  |  |  |  |
|  |  |  | European | 0.998 |  |  |  |  |
| rs192376296 | 6:32609690 | A/G | Meta | 0.081 | 1.218 (1.185-1.253)  1.124 (1.052-1.201)  1.239 (1.202-1.278) | 6.47x10^-44^  0.0005  8.51x10^-43^ | 0.009 | 3.50 |
|  |  |  | EstBB | 0.059 |  |  |  |  |
|  |  |  | FinnGen | 0.091 |  |  |  |  |
|  |  |  | European | 0.026 |  |  |  |  |
| rs76635845 | 6:33667339 | CA/C | Meta | 0.967 | 0.884 (0.846-0.924)  0.900 (0.822-0.985)  0.879 (0.836-0.924) | 3.54x10^-8^  0.023  4.66x10^-7^ | 0.657 | 0.978 |
|  |  |  | EstBB | 0.970 |  |  |  |  |
|  |  |  | FinnGen | 0.966 |  |  |  |  |
|  |  |  | European | 0.988 |  |  |  |  |
| rs9349256 | 6:43436773 | A/G | Meta | 0.541 | 1.060 (1.044-1.077)  1.046 (1.012-1.080)  1.065 (1.046-1.085) | 5.83x10^-13^  0.007  1.51x10^-11^ | 0.327 | 1.134 |
|  |  |  | EstBB | 0.522 |  |  |  |  |
|  |  |  | FinnGen | 0.550 |  |  |  |  |
|  |  |  | European | 0.485 |  |  |  |  |
| rs7764488 | 6:133491734 | A/G | Meta | 0.262 | 1.069 (1.050-1.088)  1.035 (1.000-1.073)  1.081 (1.059-1.104) | 4.57x10^-13^  0.053  3.61x10^-13^ | 0.040 | 0.784 |
|  |  |  | EstBB | 0.287 |  |  |  |  |
|  |  |  | FinnGen | 0.250 |  |  |  |  |
|  |  |  | European | 0.319 |  |  |  |  |
| rs10901863 | 10:125123701 | T/C | Meta | 0.282 | 1.103 (1.083-1.123)  1.091 (1.051-1.133)  1.106 (1.084-1.129) | 3.04x10^-27^  6.45x10^-6^  7.17x10^-23^ | 0.529 | 1.050 |
|  |  |  | EstBB | 0.257 |  |  |  |  |
|  |  |  | FinnGen | 0.294 |  |  |  |  |
|  |  |  | European | 0.280 |  |  |  |  |
| rs4922629 | 11:22832865 | T/G | Meta | 0.449 | 0.949 (0.934-0.964)  0.965 (0.934-0.997)  0.944 (0.926-0.961) | 1.30x10^-10^  0.031  7.07x10^-10^ | 0.243 | 1.064 |
|  |  |  | EstBB | 0.478 |  |  |  |  |
|  |  |  | FinnGen | 0.435 |  |  |  |  |
|  |  |  | European | 0.409 |  |  |  |  |
| rs80338939 | 13:20189546 | A/AC | Meta | 0.015 | 1.223 (1.145-1.306)  1.329 (1.193-1.480)  1.163 (1.070-1.264) | 2.20x10^-9^  2.29x10^-7^  0.0004 | 0.055 | 1.20 |
|  |  |  | EstBB | 0.022 |  |  |  |  |
|  |  |  | FinnGen | 0.012 |  |  |  |  |
|  |  |  | European | 0.010 |  |  |  |  |
| rs9532483 | 13:39970434 | T/G | Meta | 0.706 | 1.050 (1.032-1.069)  1.039 (1.002-1.077)  1.054 (1.033-1.076) | 4.40x10^-8^  0.037  3.23x10^-7^ | 0.481 | 0.997 |
|  |  |  | EstBB | 0.706 |  |  |  |  |
|  |  |  | FinnGen | 0.706 |  |  |  |  |
|  |  |  | European | 0.708 |  |  |  |  |
| rs2749882 | 14:52048541 | T/G | Meta | 0.349 | 1.058 (1.040-1.075)  1.043 (1.009-1.079)  1.062 (1.042-1.083) | 3.42x10^-11^  0.014  4.89x10^-10^ | 0.360 | 0.850 |
|  |  |  | EstBB | 0.346 |  |  |  |  |
|  |  |  | FinnGen | 0.350 |  |  |  |  |
|  |  |  | European | 0.412 |  |  |  |  |
| rs12594617 | 15:88715387 | C/G | Meta | 0.854 | 1.083 (1.058-1.109)  1.018 (0.974-1.064)  1.109 (1.079-1.140) | 1.51x10^-11^  0.436  9.86x10^-14^ | 0.001 | 1.073 |
|  |  |  | EstBB | 0.836 |  |  |  |  |
|  |  |  | FinnGen | 0.864 |  |  |  |  |
|  |  |  | European | 0.805 |  |  |  |  |
| rs1297538889 | 16:88498571 | A/G | Meta | 0.002 | 1.694 (1.452-1.976)  3.917 (2.384-6.433)  1.549 (1.317-1.821) | 2.03x10^-11^  6.98x10^-8^  1.21x10^-7^ | 0.0005 | 40.82 |
|  |  |  | EstBB | 0.001 |  |  |  |  |
|  |  |  | FinnGen | 0.003 |  |  |  |  |
|  |  |  | European | 7.4x10^-5^ |  |  |  |  |
| rs2228712 | 18:6686864 | A/C | Meta | 0.563 | 1.049 (1.032-1.066)  1.022 (0.990-1.056)  1.058 (1.038-1.078) | 5.60x10^-9^  0.178  2.73x10^-9^ | 0.073 | 1.122 |
|  |  |  | EstBB | 0.550 |  |  |  |  |
|  |  |  | FinnGen | 0.570 |  |  |  |  |
|  |  |  | European | 0.508 |  |  |  |  |
| rs2392794 | 19:472149 | T/C | Meta | 0.816 | 0.930 (0.911-0.949)  0.994 (0.953-1.037)  0.911 (0.890-0.932) | 3.12x10^-12^  0.783  5.29x10^-15^ | 0.0004 | 1.030 |
|  |  |  | EstBB | 0.814 |  |  |  |  |
|  |  |  | FinnGen | 0.817 |  |  |  |  |
|  |  |  | European | 0.793 |  |  |  |  |
| rs62128118 | 19:49602713 | A/G | Meta | 0.002 | 1.753 (1.454-2.113)  0.895 (0.658-1.219)  2.585 (2.044-3.268) | 3.93x10^-9^  0.483  2.18x10^-15^ | 8.32x10^-8^ | 0.333 |
|  |  |  | EstBB | 0.003 |  |  |  |  |
|  |  |  | FinnGen | 0.001 |  |  |  |  |
|  |  |  | European | 0.003 |  |  |  |  |
| rs78892715 | 20:61270238 | T/C | Meta | 0.110 | 1.077 (1.050-1.104)  1.091 (1.035-1.150)  1.073 (1.042-1.104) | 9.10x10^-9^  0.001  1.81x10^-6^ | 0.580 | 1.273 |
|  |  |  | EstBB | 0.105 |  |  |  |  |
|  |  |  | FinnGen | 0.112 |  |  |  |  |
|  |  |  | European | 0.088 |  |  |  |  |
| rs5756795 | 22:37726115 | T/C | Meta | 0.594 | 0.924 (0.909-0.939)  0.949 (0.920-0.980)  0.915 (0.898-0.932) | 7.71x10^-22^  0.001  1.82x10^-20^ | 0.050 | 1.124 |
|  |  |  | EstBB | 0.530 |  |  |  |  |
|  |  |  | FinnGen | 0.626 |  |  |  |  |
|  |  |  | European | 0.557 |  |  |  |  |
| rs4820434 | 22:41241115 | T/G | Meta | 0.302 | 0.953 (0.936-0.969)  0.972 (0.937-1.008)  0.947 (0.929-0.966) | 4.17x10^-8^  0.126  6.71x10^-8^ | 0.234 | 1.059 |
|  |  |  | EstBB | 0.260 |  |  |  |  |
|  |  |  | FinnGen | 0.322 |  |  |  |  |
|  |  |  | European | 0.304 |  |  |  |  |

RSID; SNP markers identification number; CHR:POS(hg38) chromosome and position; EA, effect allele; NEA, other allele; Population, study cohorts and European frequencies have been extracted form GnomAD (European (non-Finnish)); EAF; effect allele frequency; OR (95%CI) odds ratio and its 95% confidence interval; P, p-value; HetPVal, p-value of heterogeneity; Fin Enric., enrichment in Finns (calculated FinnGen EAF/European EAF, FinnGen EAF is the allele frequency in FinnGen and European EAF is the allele frequency in Europeans (does not include Finns).

## **Table S4.** The lead variants of the SNHL-associated loci (P<5x10^-8^, we defined the locus with a 1 MB window) associated in the genome-wide meta-analysis of 28 198 SNHL cases and 495 099 controls from the FinnGen and Estonian Biobank who were diagnosed at the age of 55 or older.  Loci with a grey background were located in the HLA region and were counted as one locus.

| **RSID** | **CHR:POS(hg38)** | **EA/NEA** | **Population** | **EAF** | **OR (95% CI)** | **P** | **HetPVal** | **Fin Enric** |
| --- | --- | --- | --- | --- | --- | --- | --- | --- |
| rs11211188 | 1:45789046 | A/G | Meta | 0.443 | 0.947 (0.929-0.965)  0.949 (0.929-0.969)  0.940 (0.902-0.980) | 9.22x10^-9^  7.07x10^-7^  0.0034 | 0.697 | 0.976 |
|  |  |  | FinnGen | 0.438 |  |  |  |  |
|  |  |  | EstBB | 0.451 |  |  |  |  |
|  |  |  | European | 0.449 |  |  |  |  |
| rs10176445 | 2:54618639 | C/G | Meta | 0.186 | 1.116 (1.090-1.142)  1.130 (1.100-1.160)  1.065 (1.013-1.121) | 5.22x10^-20^  1.39x10^-19^  0.015 | 0.045 | 0.784 |
|  |  |  | FinnGen | 0.178 |  |  |  |  |
|  |  |  | EstBB | 0.202 |  |  |  |  |
|  |  |  | European | 0.227 |  |  |  |  |
| rs9820091 | 3:156549608 | T/C | Meta | 0.861 | 1.085 (1.056-1.115)  1.091 (1.058-1.125)  1.064 (1.004-1.126) | 4.22x10^-9^  3.10x10^-8^  0.035 | 0.440 | 1.038 |
|  |  |  | FinnGen | 0.868 |  |  |  |  |
|  |  |  | EstBB | 0.848 |  |  |  |  |
|  |  |  | European | 0.836 |  |  |  |  |
| rs116773267 | 5:51156672 | A/G | Meta | 0.936 | 1.122 (1.080-1.165)  1.121 (1.077-1.167)  1.126 (1.008-1.257) | 2.27x10^-9^  2.24x10^-8^  0.035 | 0.942 | 0.941 |
|  |  |  | FinnGen | 0.924 |  |  |  |  |
|  |  |  | EstBB | 0.960 |  |  |  |  |
|  |  |  | European | 0.982 |  |  |  |  |
| rs2161095 | 5:73656208 | T/C | Meta | 0.482 | 0.942 (0.925-0.960)  0.940 (0.921-0.960)  0.951 (0.913-0.992) | 3.25x10^-10^  4.99x10^-9^  0.018 | 0.614 | 1.105 |
|  |  |  | FinnGen | 0.474 |  |  |  |  |
|  |  |  | EstBB | 0.497 |  |  |  |  |
|  |  |  | European | 0.429 |  |  |  |  |
| rs187576526 | 6:26336881 | T/C | Meta | 0.016 | 1.217 (1.136-1.304)  1.231 (1.148-1.321)  0.961 (0.701-1.319) | 2.21x10^-8^  7.16x10^-9^  0.806 | 0.134 | 55.00 |
|  |  |  | FinnGen | 0.022 |  |  |  |  |
|  |  |  | EstBB | 0.004 |  |  |  |  |
|  |  |  | European | 0.0004 |  |  |  |  |
| rs181707255 | 6:27396159 | T/C | Meta | 0.018 | 1.261 (1.183-1.344)  1.274 (1.193-1.360)  0.994 (0.727-1.359) | 1.24x10^-12^  3.81x10^-13^  0.969 | 0.128 | 50.00 |
|  |  |  | FinnGen | 0.025 |  |  |  |  |
|  |  |  | EstBB | 0.005 |  |  |  |  |
|  |  |  | European | 0.0005 |  |  |  |  |
| rs149383874 | 6:28706589 | A/G | Meta | 0.982 | 0.804 (0.754-0.857)  0.796 (0.746-0.850)  0.984 (0.727-1.331) | 1.79x10^-11^  7.21x10^-12^  0.917 | 0.180 | 0.976 |
|  |  |  | FinnGen | 0.975 |  |  |  |  |
|  |  |  | EstBB | 0.995 |  |  |  |  |
|  |  |  | European | 0.999 |  |  |  |  |
| rs144200919 | 6:29747323 | A/G | Meta | 0.017 | 1.237 (1.158-1.322)  1.248 (1.166-1.336)  1.035 (0.764-1.403) | 3.32x10^-10^  1.67x10^-10^  0.824 | 0.239 | 46.00 |
|  |  |  | FinnGen | 0.023 |  |  |  |  |
|  |  |  | EstBB | 0.005 |  |  |  |  |
|  |  |  | European | 0.0005 |  |  |  |  |
| rs190777367 | 6:30939492 | T/G | Meta | 0.981 | 0.793 (0.744-0.844)  0.775 (0.726-0.828)  1.016 (0.814-1.267) | 4.53x10^-13^  3.19x10^-14^  0.891 | 0.022 | 0.978 |
|  |  |  | FinnGen | 0.976 |  |  |  |  |
|  |  |  | EstBB | 0.991 |  |  |  |  |
|  |  |  | European | 0.998 |  |  |  |  |
| rs192376296 | 6:32609690 | A/G | Meta | 0.080 | 1.214 (1.175-1.254)  1.224 (1.182-1.267)  1.157 (1.063-1.260) | 5.04x10^-32^  7.24x10^-30^  0.00078 | 0.231 | 3.033 |
|  |  |  | FinnGen | 0.091 |  |  |  |  |
|  |  |  | EstBB | 0.059 |  |  |  |  |
|  |  |  | European | 0.030 |  |  |  |  |
| rs2242416 | 6:43305866 | A/G | Meta | 0.369 | 0.933 (0.915-0.951)  0.924 (0.904-0.944)  0.965 (0.926-1.007) | 1.02x10^-12^  7.53x10^-13^  0.102 | 0.070 | 0.875 |
|  |  |  | FinnGen | 0.356 |  |  |  |  |
|  |  |  | EstBB | 0.396 |  |  |  |  |
|  |  |  | European | 0.407 |  |  |  |  |
| rs7764488 | 6:133491734 | A/G | Meta | 0.262 | 1.080 (1.057-1.103)  1.080 (1.055-1.106)  1.079 (1.032-1.129) | 7.51x10^-13^  2.08x10^-10^  0.00090 | 0.981 | 0.784 |
|  |  |  | FinnGen | 0.250 |  |  |  |  |
|  |  |  | EstBB | 0.287 |  |  |  |  |
|  |  |  | European | 0.319 |  |  |  |  |
| rs12772368 | 10:69811134 | T/C | Meta | 0.158 | 1.074 (1.047-1.101)  1.087 (1.056-1.119)  1.028 (0.974-1.085) | 3.99x10^-8^  1.30x10^-8^  0.310 | 0.0734 | 0.907 |
|  |  |  | FinnGen | 0.147 |  |  |  |  |
|  |  |  | EstBB | 0.179 |  |  |  |  |
|  |  |  | European | 0.162 |  |  |  |  |
| rs7909581 | 10:92953298 | T/C | Meta | 0.347 | 1.059 (1.039-1.080)  1.060 (1.038-1.083)  1.055 (1.010-1.101) | 6.04x10^-9^  1.20x10^-7^  0.016 | 0.834 | 1.085 |
|  |  |  | FinnGen | 0.345 |  |  |  |  |
|  |  |  | EstBB | 0.352 |  |  |  |  |
|  |  |  | European | 0.318 |  |  |  |  |
| rs10901863 | 10:125123701 | T/C | Meta | 0.281 | 1.113 (1.090-1.136)  1.113 (1.088-1.139)  1.111 (1.058-1.167) | 2.06x10^-24^  1.84x10^-20^  2.20x10^-5^ | 0.953 | 1.050 |
|  |  |  | FinnGen | 0.294 |  |  |  |  |
|  |  |  | EstBB | 0.256 |  |  |  |  |
|  |  |  | European | 0.280 |  |  |  |  |
| rs4922629 | 11:22832865 | T/G | Meta | 0.450 | 0.946 (0.928-0.964)  0.944 (0.924-0.963)  0.954 (0.915-0.995) | 4.43x10^-9^  4.68x10^-8^  0.028 | 0.633 | 1.064 |
|  |  |  | FinnGen | 0.435 |  |  |  |  |
|  |  |  | EstBB | 0.478 |  |  |  |  |
|  |  |  | European | 0.409 |  |  |  |  |
| rs9532483 | 13:39970434 | T/G | Meta | 0.706 | 1.061 (1.039-1.083)  1.062 (1.038-1.087)  1.057 (1.009-1.106) | 1.43x10^-8^  2.44x10^-7^  0.019 | 0.847 | 0.997 |
|  |  |  | FinnGen | 0.706 |  |  |  |  |
|  |  |  | EstBB | 0.706 |  |  |  |  |
|  |  |  | European | 0.708 |  |  |  |  |
| rs1566128 | 14:52048263 | A/G | Meta | 0.349 | 1.070 (1.050-1.091)  1.072 (1.049-1.095)  1.066 (1.021-1.114) | 3.90x10^-12^  2.85x10^-10^  0.0037 | 0.846 | 0.850 |
|  |  |  | FinnGen | 0.350 |  |  |  |  |
|  |  |  | EstBB | 0.347 |  |  |  |  |
|  |  |  | European | 0.412 |  |  |  |  |
| rs12372879 | 14:64673708 | A/G | Meta | 0.358 | 1.059 (1.039-1.079)  1.053 (1.031-1.076)  1.081 (1.036-1.128) | 7.01x10^-9^  2.79x10^-6^  0.00036 | 0.287 | 0.902 |
|  |  |  | FinnGen | 0.351 |  |  |  |  |
|  |  |  | EstBB | 0.372 |  |  |  |  |
|  |  |  | European | 0.389 |  |  |  |  |
| rs34295421 | 15:88708947 | C/G | Meta | 0.838 | 1.098 (1.070-1.126)  1.117 (1.085-1.150)  1.032 (0.977-1.090) | 1.37x10^-12^  1.12x10^-13^  0.258 | 0.013 | 1.089 |
|  |  |  | FinnGen | 0.847 |  |  |  |  |
|  |  |  | EstBB | 0.821 |  |  |  |  |
|  |  |  | European | 0.778 |  |  |  |  |
| rs1297538889 | 16:88498571 | A/G | Meta | 0.002 | 1.743 (1.464-2.075)  1.606 (1.339-1.925)  4.722 (2.512-8.879) | 4.20x10^-10^  3.14x10^-7^  1.44x10^-6^ | 0.001 | 40.82 |
|  |  |  | FinnGen | 0.003 |  |  |  |  |
|  |  |  | EstBB | 0.001 |  |  |  |  |
|  |  |  | European | 7.4x10^-5^ |  |  |  |  |
| rs199827975 | 19:460292 | T/TC | Meta | 0.117 | 1.101 (1.070-1.133)  1.115 (1.080-1.151)  1.050 (0.986-1.118) | 3.72x10^-11^  2.86x10^-11^  0.131 | 0.095 | 0.927 |
|  |  |  | FinnGen | 0.114 |  |  |  |  |
|  |  |  | EstBB | 0.123 |  |  |  |  |
|  |  |  | European | 0.123 |  |  |  |  |
| rs199600574 | 19:50301724 | T/C | Meta | 0.014 | 1.239 (1.152-1.332)  1.248 (1.159-1.344)  1.073 (0.775-1.486) | 6.70x10^-9^  4.90x10^-9^  0.672 | 0.375 | 36.00 |
|  |  |  | FinnGen | 0.018 |  |  |  |  |
|  |  |  | EstBB | 0.005 |  |  |  |  |
|  |  |  | European | 0.0005 |  |  |  |  |
| rs5756795 | 22:37726115 | T/C | Meta | 0.594 | 0.913 (0.896-0.930) | 2.35x10^-21^ | 0.198 | 1.124 |
|  |  |  | FinnGen | 0.626 | 0.907 (0.888-0.926) | 1.78x10^-19^ |  |  |
|  |  |  | EstBB | 0.530 | 0.935 (0.897-0.974) | 0.0014 |  |  |
|  |  |  | European | 0.557 |  |  |  |  |

RSID; SNP markers identification number; CHR:POS(hg38) chromosome and position; EA, effect allele; NEA, other allele; Population, study cohorts and European frequencies have been extracted form GnomAD (European (non-Finnish)); EAF; effect allele frequency; OR (95%CI) odds ratio and its 95% confidence interval; P, p-value; HetPVal, p-value of heterogeneity; Fin Enric., enrichment in Finns (calculated FinnGen EAF/European EAF, FinnGen EAF is the allele frequency in FinnGen and European EAF is the allele frequency in Europeans (does not include Finns).

**Table S5.** The lead variants of the SNHL-associated loci (P<5x10^-8^, we defined the locus with a 1 MB window) associated in the genome-wide meta-analysis of 7762 SNHL cases and 495 099 controls from the FinnGen and Estonian Biobank who were diagnosed before the age of 55.  Locus with a grey background were located in the HLA region.

| **RSID** | **CHR:POS (hg38)** | **EA/NEA** | **Population** | **EAF** | **OR (95% CI)** | **P** | **HetPVal** | **Fin Enric.** |
| --- | --- | --- | --- | --- | --- | --- | --- | --- |
| rs116239570 | 6:32376554 | A/G | Meta | 0.082 | 1.250 (1.184-1.321)  1.296 (1.215-1.382)  1.142 (1.030-1.266) | 1.10x10^-15^  3.49x10^-15^  0.0113 | 0.042 | 2.818 |
|  |  |  | FinnGen | 0.093 |  |  |  |  |
|  |  |  | EstBB | 0.064 |  |  |  |  |
|  |  |  | European | 0.033 |  |  |  |  |
| rs80338939 | 13:20189546 | A/AC | Meta | 0.015 | 1.749 (1.562-1.960)  1.852 (1.579-2.173)  1.651 (1.405-1.939) | 4.16x10^-22^  3.79x10^-14^  1.09x10^-9^ | 0.320 | 1.200 |
|  |  |  | FinnGen | 0.012 |  |  |  |  |
|  |  |  | EstBB | 0.022 |  |  |  |  |
|  |  |  | European | 0.010 |  |  |  |  |
| rs182243786 | 18:39882914 | A/T | Meta | 0.993 | 0.616 (0.518-0.732)  0.662 (0.527-0.830)  0.558 (0.428-0.729) | 3.81x10^-8^  0.00036  1.77x10^-5^ | 0.341 | 0.999 |
|  |  |  | FinnGen | 0.994 |  |  |  |  |
|  |  |  | EstBB | 0.992 |  |  |  |  |
|  |  |  | European | 0.995 |  |  |  |  |

RSID; SNP markers identification number; CHR:POS(hg38) chromosome and position; EA, effect allele; NEA, other allele; Population, study cohorts and European frequencies have been extracted form GnomAD (European (non-Finnish)); EAF; effect allele frequency; OR (95%CI) odds ratio and its 95% confidence interval; P, p-value; HetPVal, p-value of heterogeneity; Fin Enric., enrichment in Finns (calculated FinnGen EAF/European EAF, FinnGen EAF is the allele frequency in FinnGen and European EAF is the allele frequency in Europeans (does not include Finns).

## **Table S6.** The lead variants of the SNHL-associated loci (P<5x10^-8^, we defined the locus with a 1 MB window) associated in the genome-wide meta-analysis of 17 761 SNHL female cases and 295 740 same-sex controls from the FinnGen and Estonian Biobank.  Loci with a grey background were located in the HLA region and were counted as one locus.

| **RSID** | **CHR:POS (hg38)** | **EA/NEA** | **Population** | **EAF** | | **OR (95% CI)** | **P** | **Het**  **Pval** | **FIn Enric.** |
| --- | --- | --- | --- | --- | --- | --- | --- | --- | --- |
| rs10176445 | 2:54618639 | C/G | Meta | 0.186 | 1.103 (1.072-1.135)  1.117 (1.080-1.156)  1.072 (1.018-1.128) | | 1.36x10^-11^  2.04x10^-10^  0.0080 | 0.189 | 0.780 |
|  |  |  | FinnGen | 0.177 |  |  |  |  |  |
|  |  |  | EstBB | 0.202 |  |  |  |  |  |
|  |  |  | European | 0.227 |  |  |  |  |  |
| rs7640872 | 3:121978321 | A/C | Meta | 0.254 | 1.078 (1.051-1.106)  1.093 (1.060-1.126)  1.043 (0.995-1.094) | | 8.43x10^-9^  1.10x10^-8^  0.080 | 0.111 | 0.973 |
|  |  |  | FinnGen | 0.251 |  |  |  |  |  |
|  |  |  | EstBB | 0.258 |  |  |  |  |  |
|  |  |  | European | 0.258 |  |  |  |  |  |
| rs4704097 | 5:73773756 | A/G | Meta | 0.465 | 0.920 (0.899-0.941)  0.909 (0.885-0.934)  0.947 (0.907-0.989) | | 5.65x10^-13^  3.60x10^-12^  0.013 | 0.111 | 1.009 |
|  |  |  | FinnGen | 0.457 |  |  |  |  |  |
|  |  |  | EstBB | 0.481 |  |  |  |  |  |
|  |  |  | European | 0.453 |  |  |  |  |  |
| rs181556321 | 6:27734302 | T/C | Meta | 0.031 | 1.205 (1.131-1.283)  1.229 (1.146-1.318)  1.112 (0.967-1.279) | | 6.04x10^-9^  8.34x10^-9^  0.138 | 0.210 | 5.00 |
|  |  |  | FinnGen | 0.035 |  |  |  |  |  |
|  |  |  | EstBB | 0.023 |  |  |  |  |  |
|  |  |  | European | 0.007 |  |  |  |  |  |
| rs9257453 | 6:29001153 | A/G | Meta | 0.873 | 0.911 (0.882-0.942)  0.913 (0.878-0.948)  0.908 (0.851-0.969) | | 3.44x10^-8^  2.85x10^-6^  0.0035 | 0.889 | 0.941 |
|  |  |  | FinnGen | 0.864 |  |  |  |  |  |
|  |  |  | EstBB | 0.887 |  |  |  |  |  |
|  |  |  | European | 0.918 |  |  |  |  |  |
| rs2517600 | 6:30106386 | A/G | Meta | 0.281 | 0.929 (0.905-0.953)  0.929 (0.902-0.957)  0.927 (0.879-0.978) | | 2.51x10^-8^  1.39x10^-6^  0.005 | 0.940 | 1.171 |
|  |  |  | FinnGen | 0.287 |  |  |  |  |  |
|  |  |  | EstBB | 0.270 |  |  |  |  |  |
|  |  |  | European | 0.245 |  |  |  |  |  |
| rs191912046 | 6:31498278 | A/T | Meta | 0.017 | 1.290 (1.188-1.401) | | 1.42x10^-9^ | 0.391 | 2.222 |
|  |  |  | FinnGen | 0.020 | 1.311 (1.198-1.435) | | 4.16x10^-9^ |  |  |
|  |  |  | EstBB | 0.011 | 1.190 (0.972-1.457) | | 0.093 |  |  |
|  |  |  | European | 0.009 |  | |  |  |  |
| rs192376296 | 6:32609690 | A/G | Meta | 0.080 | 1.359 (1.308-1.413)  1.415 (1.355-1.477)  1.160 (1.064-1.264) | | 1.22x10^-54^  1.00x10^-55^  0.00074 | 5.2x10^-5^ | 3.538 |
|  |  |  | FinnGen | 0.092 |  |  |  |  |  |
|  |  |  | EstBB | 0.059 |  |  |  |  |  |
|  |  |  | European | 0.026 |  |  |  |  |  |
| rs9369409 | 6:43378724 | C/G | Meta | 0.382 | 0.936 (0.914-0.958)  0.918 (0.893-0.944)  0.979 (0.938-1.022) | | 2.59x10^-8^  1.77x10^-9^  0.336 | 0.013 | 0.880 |
|  |  |  | FinnGen | 0.368 |  |  |  |  |  |
|  |  |  | EstBB | 0.407 |  |  |  |  |  |
|  |  |  | European | 0.418 |  |  |  |  |  |
| rs146694394 | 6:158071628 | T/C | Meta | 0.003 | 1.742 (1.451-2.091)  1.681 (1.304-2.166)  1.810 (1.391-2.355) | | 2.60x10^-9^  6.08x10^-5^  9.82x10^-6^ | 0.691 | 0.400 |
|  |  |  | FinnGen | 0.002 |  |  |  |  |  |
|  |  |  | EstBB | 0.005 |  |  |  |  |  |
|  |  |  | European | 0.005 |  |  |  |  |  |
| rs10901863 | 10:125123701 | T/C | Meta | 0.280 | 1.117 (1.089-1.145)  1.121 (1.088-1.154)  1.106 (1.053-1.162) | | 6.54x10^-18^  2.08x10^-14^  6.05x10^-5^ | 0.663 | 1.050 |
|  |  |  | FinnGen | 0.294 |  |  |  |  |  |
|  |  |  | EstBB | 0.257 |  |  |  |  |  |
|  |  |  | European | 0.280 |  |  |  |  |  |
| rs4922629 | 11:22832865 | T/G | Meta | 0.451 | 0.938 (0.917-0.959)  0.938 (0.913-0.963)  0.937 (0.898-0.978) | | 3.05x10^-8^  3.07x10^-6^  0.0028 | 0.982 | 1.064 |
|  |  |  | FinnGen | 0.435 |  |  |  |  |  |
|  |  |  | EstBB | 0.477 |  |  |  |  |  |
|  |  |  | European | 0.409 |  |  |  |  |  |
| rs12793619 | 11:77210313 | A/G | Meta | 0.372 | 0.935 (0.913-0.957)  0.925 (0.900-0.951)  0.962 (0.919-1.006) | | 2.64x10^-8^  3.91x10^-8^  0.091 | 0.148 | 1.343 |
|  |  |  | FinnGen | 0.388 |  |  |  |  |  |
|  |  |  | EstBB | 0.344 |  |  |  |  |  |
|  |  |  | European | 0.289 |  |  |  |  |  |
| rs80338939 | 13:20189546 | A/AC | Meta | 0.015 | 1.299 (1.186-1.422)  1.175 (1.041-1.325)  1.481 (1.291-1.700) | | 1.59x10^-8^  0.0088  2.28x10^-8^ | 0.013 | 1.200 |
|  |  |  | FinnGen | 0.012 |  |  |  |  |  |
|  |  |  | EstBB | 0.022 |  |  |  |  |  |
|  |  |  | European | 0.010 |  |  |  |  |  |
| rs7143885 | 14:103725580 | C/G | Meta | 0.399 | 1.071 (1.047-1.096)  1.070 (1.041-1.100)  1.072 (1.028-1.119) | | 5.02x10^-9^  1.03x10^-6^  0.0013 | 0.936 | 1.147 |
|  |  |  | FinnGen | 0.399 |  |  |  |  |  |
|  |  |  | EstBB | 0.398 |  |  |  |  |  |
|  |  |  | European | 0.348 |  |  |  |  |  |
| rs1267664387 | 16:88658954 | A/C | Meta | 0.002 | 1.821 (1.489-2.226)  1.731 (1.395-2.148)  2.542 (1.461-4.422) | | 5.21x10^-9^  6.34x10^-7^  0.00096 | 0.205 | 5.00 |
|  |  |  | FinnGen | 0.003 |  |  |  |  |  |
|  |  |  | EstBB | 0.001 |  |  |  |  |  |
|  |  |  | European | 0.0006 |  |  |  |  |  |
| rs2392794 | 19:472149 | T/C | Meta | 0.816 | 0.917 (0.891-0.944)  0.909 (0.879-0.941)  0.937 (0.887-0.989) | | 4.16x10^-9^  4.81x10^-8^  0.018 | 0.370 | 1.030 |
|  |  |  | FinnGen | 0.817 |  |  |  |  |  |
|  |  |  | EstBB | 0.814 |  |  |  |  |  |
|  |  |  | European | 0.793 |  |  |  |  |  |
| rs78892715 | 20:61270238 | T/C | Meta | 0.109 | 1.106 (1.067-1.146)  1.098 (1.054-1.145)  1.125 (1.051-1.205) | | 3.25x10^-8^  1.01x10^-5^  0.00074 | 0.561 | 1.273 |
|  |  |  | FinnGen | 0.112 |  |  |  |  |  |
|  |  |  | EstBB | 0.105 |  |  |  |  |  |
|  |  |  | European | 0.088 |  |  |  |  |  |
| rs5756795 | 22:37726115 | T/C | Meta | 0.591 | 0.893 (0.873-0.914)  0.878 (0.855-0.903)  0.928 (0.890-0.967) | | 2.23x10^-22^  1.09x10^-20^  0.00044 | 0.031 | 1.124 |
|  |  |  | FinnGen | 0.626 |  |  |  |  |  |
|  |  |  | EstBB | 0.531 |  |  |  |  |  |
|  |  |  | European | 0.557 |  |  |  |  |  |

RSID; SNP markers identification number; CHR:POS(hg38) chromosome and position; EA, effect allele; NEA, other allele; Population, study cohorts and European frequencies have been extracted form GnomAD (European (non-Finnish)); EAF; effect allele frequency; OR (95%CI) odds ratio and its 95% confidence interval; P, p-value; HetPVal, p-value of heterogeneity; Fin Enric., enrichment in Finns (calculated FinnGen EAF/European EAF, FinnGen EAF is the allele frequency in FinnGen and European EAF is the allele frequency in Europeans (does not include Finns).

## **Table S7.** The lead variants of the SNHL-associated loci (P<5x10^-8^, we defined the locus with a 1 MB window) associated in the genome-wide meta-analysis of 18 199 SNHL male cases and 199 359 same-sex controls from the FinnGen and Estonian Biobank.

| **RSID** | **CHR:POS (hg38)** | **EA/NEA** | **Population** | **EAF** | **OR (95% CI)** | **P** | **HetPVal** | **Fin Enric.** |
| --- | --- | --- | --- | --- | --- | --- | --- | --- |
| rs7608414 | 2:54617858 | A/G | Meta | 0.187 | 1.091 (1.060-1.123)  1.114 (1.078-1.150)  1.009 (0.947-1.075) | 2.69x10^-9^  6.47x10^-11^  0.776 | 0.007 | 0.786 |
|  |  |  | FinnGen | 0.180 |  |  |  |  |
|  |  |  | EstBB | 0.203 |  |  |  |  |
|  |  |  | European | 0.229 |  |  |  |  |
| rs10901863 | 10:125123701 | T/C | Meta | 0.283 | 1.091 (1.064-1.119)  1.095 (1.065-1.125)  1.074 (1.011-1.141) | 1.20x10^-11^  1.63x10^-10^  0.020 | 0.577 | 1.050 |
|  |  |  | FinnGen | 0.294 |  |  |  |  |
|  |  |  | EstBB | 0.255 |  |  |  |  |
|  |  |  | European | 0.280 |  |  |  |  |
| rs756330 | 13:78781316 | A/G | Meta | 0.905 | 0.892 (0.859-0.926)  0.892 (0.854-0.932)  0.890 (0.824-0.962) | 3.45x10^-9^  3.01x10^-7^  0.003 | 0.962 | 1.046 |
|  |  |  | FinnGen | 0.913 |  |  |  |  |
|  |  |  | EstBB | 0.883 |  |  |  |  |
|  |  |  | European | 0.873 |  |  |  |  |
| rs987473446 | 19:48828523 | C/G | Meta | 0.9995 | 0.319 (0.212-0.478) | 3.29x10^-8^ | 0.252 | 0.999 |
|  |  |  | FinnGen | 0.9993 | 0.311 (0.207-0.467) | 1.97x10^-8^ |  |  |
|  |  |  | EstBB | 0.9998 | 3.053 (0.063-148) | 0.574 |  |  |
|  |  |  | European | 0.9999 |  |  |  |  |

RSID; SNP markers identification number; CHR:POS(hg38) chromosome and position; EA, effect allele; NEA, other allele; Population, study cohorts and European frequencies have been extracted form GnomAD (European (non-Finnish)); EAF; effect allele frequency; OR (95%CI) odds ratio and its 95% confidence interval; P, p-value; HetPVal, p-value of heterogeneity; Fin Enric., enrichment in Finns (calculated FinnGen EAF/European EAF, FinnGen EAF is the allele frequency in FinnGen and European EAF is the allele frequency in Europeans (does not include Finns).

**References**

1.	Orvis, J. *et al.* gEAR: Gene Expression Analysis Resource portal for community-driven, multi-omic data exploration. *Nat. Methods* **18**, 843–844 (2021).

2.	van der Valk, W. H. *et al.* A single-cell level comparison of human inner ear organoids with the human cochlea and vestibular organs. *Cell Rep.* **42**, 112623 (2023).

3.	Giambartolomei, C. *et al.* Bayesian test for colocalisation between pairs of genetic association studies using summary statistics. *PLoS Genet.* **10**, e1004383 (2014).

4.	Sliz, E. *et al.* Evidence of a causal effect of genetic tendency to gain muscle mass on uterine leiomyomata. *Nat. Commun.* **14**, 542 (2023).
